# Computer-aided identification of potential inhibitors against *Necator americanus* glutathione *S*-transferase 3

**DOI:** 10.1016/j.imu.2022.100957

**Published:** 2022

**Authors:** Samuel K. Kwofie, Seth O. Asiedu, Russell Koranteng, Emelia Quarshie, Elvis K. Tiburu, Whelton A. Miller, Michael B. Adinortey, Michael D. Wilson

**Affiliations:** a Department of Biomedical Engineering, School of Engineering Sciences, College of Basic and Applied Sciences, University of Ghana, Legon, Accra, Ghana; b West African Center for Cell Biology and Infectious Pathogens, Department of Biochemistry, Cell and Molecular Biology, University of Ghana, Legon, Accra, Ghana; c Department of Parasitology, Noguchi Memorial Institute for Medical Research (NMIMR), College of Health Sciences (CHS), University of Ghana, Legon, Accra, Ghana; d Department of Medicine, Loyola University Medical Center, Maywood, IL, 60153, USA; e Department of Chemical and Biomolecular Engineering, School of Engineering and Applied Science, University of Pennsylvania, Philadelphia, PA, USA; f Department of Molecular Pharmacology and Neuroscience, Loyola University Medical Center, Maywood, IL, 60153, USA; g Department of Biochemistry, School of Biological Sciences, College of Agricultural and Natural Sciences, University of Cape Coast, Ghana

**Keywords:** *Necator americanus*, Natural products, Pharmacoinformatics, Molecular dynamics, Molecular docking, Anthelmintics

## Abstract

Hookworm infection is caused by the blood-feeding hookworm gastrointestinal nematodes. Its harmful effects include anemia and retarded growth and are common in the tropics. A current control method involves the mass drug administration of synthetic drugs, mainly albendazole and mebendazole. There are however concerns of low efficacy and drug resistance due to their repeated and excessive use. Although, *Necator americanus* glutathione *S*-transferase 3 (*Na*-GST-3) is a notable target, using natural product libraries for computational elucidation of promising leads is underexploited. This study sought to use pharmacoinformatics techniques to identify compounds of natural origins with the potential to be further optimized as promising inhibitors. A compendium of 3182 African natural products together with five known helminth GST inhibitors including Cibacron blue was screened against the active sites of the *Na*-GST-3 structure (PDB ID: 3W8S). The hit compounds were profiled to ascertain the mechanisms of binding, anthelmintic bioactivity, physicochemical and pharmacokinetic properties. The AutoDock Vina docking protocol was validated by obtaining 0.731 as the area under the curve calculated *via* the receiver operating characteristics curve. Four compounds comprising ZINC85999636, ZINC35418176, ZINC14825190, and Dammarane Triterpene13 were identified as potential lead compounds with binding energies less than −9.0 kcal/mol. Furthermore, the selected compounds formed key intermolecular interactions with critical residues Tyr95, Gly13 and Ala14. Notably, ZINC85999636, ZINC14825190, and dammarane triterpene13 were predicted as anthelmintics, whilst all the four molecules shared structural similarities with known inhibitors. Molecular modelling showed that the compounds had reasonably good binding free energies. More so, they had high binding affinities when screened against other variants of the *Na*-GST, namely *Na*-GST-1 and *Na*-GST-2. Ligand quality assessment using ligand efficiency dependent lipophilicity, ligand efficiency, ligand efficiency scale and fit quality scale showed the molecules are worthy candidates for further optimization. The inhibitory potentials of the molecules warrant *in vitro* studies to evaluate their effect on the heme regulation mechanisms.

## 1. Introduction

Hookworm infection affects over 500 million people globally [1,2]. Due to its debilitating effects such as chronic anemia, diarrhea, and retarded growth in children, hookworm infection has been labelled “the great infection of mankind” [3,4]. Hookworm infection is a neglected tropical disease that affect over a billion people and costs both developing and underdeveloped economies billions of dollars annually but unfortunately has not been given the much-needed attention [1]. Hookworm infection is prevalent in tropical and sub-tropical climates. *Necator americanus*, one of the two main hookworm species, accounts for over 80% of reported hookworm infection cases in sub-Saharan Africa [1]. Ghana, a developing country in the tropics, had an estimated prevalence of 2,993,431 in 2017 [5].

Current hookworm intervention includes the mass drug administration (MDA) of the commonly used drugs benzimidazole derivatives mebendazole and albendazole [1]. Both drugs bind to the β-tubulin of the parasite and interrupt the parasite’s metabolism, slowly resulting in their death [6,7]. Binding to the nematode’s β-tubulin inhibits polymerization, thereby preventing the formation of microtubules and so stopping cell division [8]. Although, this intervention has led to some degree of success in the past [9], there are increasing reports of respondence to treatment [10–14] which has been linked to mutations in β-tubulin [15].

Therefore, new drugs with different modes of binding and mechanisms of action are urgently needed to circumvent the concerns of mutations in β-tubulin [16]. Hence, new receptors must be explored to identify a novel class of biotherapeutics. Since exogenous heme is needed by homoproteins [17], the hookworm employs the glutathione *S*-transferase (GST) family to detoxify and transport the toxic heme. By pairing to form a homodimer, the GST creates a specific pocket capable of binding the heme [18]. Inhibiting the glutathione *S*-transferase 3 of *Necator americanus* (*Na*-GST-3*)* will deprive the parasite of its major detoxification system [19] with possible death. Therefore, *Na*-GST-3 is considered a druggable target [19–21], warranting its use in this study. It plays a role in detoxifying toxic reactive oxidative species (ROS) [19]. Blood of the host containing haemoglobin enters the lumen of the hookworm, further undergoes a cascade of mechanisms which finally results in the release of heme. The heme produced is ROS, hence toxic to the hookworm [18]. The nematode-specific nu-class GSTs act as heme regulators [22]. A key distinguishing factor between nematode-specific nu-class GST and other GSTs, including humans, is the flexibility at the H-site, resulting in different substrate specificity [19]. Moreover, *Na*-GST-3 has a wider binding cavity relative to the other *Na*-GSTs, enabling it to bind to a wider array of ligands [19].

Natural products have served as treatment options for helminth infections [23,24]. They are chemically diverse and are naturally bioactive [25]. Current drug discovery efforts employ the use of natural products as scaffolds [26]. The conventional methods used in the drug discovery pipeline are expensive and time-consuming [27]. Pharmacoinformatics has evolved as an alternative method for drug discovery because it is cheaper and requires a lesser amount of time [28].

Our goal was to utilize pharmacoinformatics-based techniques to unravel novel compounds with propensity to disrupt the *Na*-GST-3 heme regulation process. The study sought to virtually screen a library of diverse compounds originating from Africa’s vast biodiversity in addition to standard helminth GST inhibitors against *Na*-GST-3. We also sought to probe into the mechanism of binding as well as to characterize computationally the anthelmintic propensity and the pharmacological profiles of these compounds. The molecular mechanics Poisson-Boltzmann surface area (MM-PBSA) approach was used to assess the binding free energies and the energy contributions of the protein-ligand complexes and residues, respectively [29]. Additionally, we screened selected molecules against other *Na*-GSTs to understand their mechanisms of binding.

## 2. Materials and methods

### 2.1. Retrieval of protein structure and molecular dynamics (MD) simulation

The solved protein structure of *Na*-GST-3 (ID: 3W8S) [19] was obtained *via* the Protein Data Bank [30] in a pdb file format. The structure was preprocessed and visualized using PyMOL 2.0.0 [31]. The structure is preprocessed by removing all available water molecules and ligands. An MD simulation of the *Na*-GST-3 target structure was performed in the GROMACS v.2018 [32]. Position restrain files and protein topology were calculated using GROMOS96 43a1 force field. The protein structure was subjected to Periodic Boundary Conditions (PBC) centered at 1 nm [33]. SPC water and 2Na^+^ were used for solvation and neutralization, respectively. The steps used for minimization of energy were 50,000 *via* the steepest descent. Equilibration was undertaken at 100 ps with 1 bar pressure and 300 K temperature *via* ensembles of NPT and NVT. The molecular dynamics production run was then performed for 100 ns and Xmgrace was used to generate output graphs [34].

### 2.2. Validation docking parameters

To determine the accuracy of the AutoDock Vina [35] docking protocol, two methods were employed. The first comprises the superimposition of the re-docked and co-crystallized ligands and the second is the generation of receiver operating characteristic (ROC) curves to assess AutoDock Vina. Glutathione (GSH), which binds to the G-site at the highly conserved N-terminal was extracted from the crystallographic structure of *Na*-GST-3, which was then re-docked to the G-site. The lowest energy pose of GSH was used in superimposition studies by juxtaposing it to the original crystallographic GSH. The root-mean-square deviation (RMSD) was calculated using LigAlign [36].

Additionally, to assess the effectiveness of Autodock Vina to distinguish between decoys and active compounds, the receiver operating characteristic (ROC) curves were produced [37]. The conventional inhibitors Cibacron blue and lithocholic acid in addition to other helminth GST inhibitors comprising ellagic acid, alizarin, and chenodeoxycholic acid [38] were used as actives. These compounds were used as inputs to generate decoys with similar physicochemical properties using DUD-E, a Directory of Useful Decoys [39]. For one inhibitor, 50 decoys were generated, but forty-five were used for screening after eliminating redundancy. A library of 230 compounds comprising 5 actives and 225 decoys were screened against the *Na*-GST-3, *Na*-GST-1 and *Na*-GST-2. Screening Explorer [40] was used to generate the ROC Curve.

### 2.3. Ligand preparations ands and molecular docking

Molecular docking of the natural compounds against the energy minimized *Na*-GST-3 was undertaken using AutoDock Vina [35] accessible *via* PyRx [41]. The 3182 African natural compounds were accessed from the AfroDb [42], a catalogue of the ZINC database [43,44], and the Northern African Natural Products Database (NANPDB) [45]. The compounds were filtered *via* the FAFDrugs4 [46] to eliminate compounds with undesirable functional groups. Additionally, known helminth GST inhibitors namely Cibacron blue, ellagic acid, alizarin, chenodeoxycholic acid, and lithocholic acid [38] were also included in the screening set. The OpenBabel [47] was used for preparing the compounds, and 200 steps were used for the Universal Force Field (UFF) and optimization was undertaken using the conjugate gradient. A grid box was specified mainly around the ligand binding H-site with dimension (30.578-X, 20.762-Y, 38.733-Z) Å and centered at (44.396, 40.678, 40.164) Å. The ligands were docked to the protein with a default exhaustiveness of 8. The top 5% compounds were then evaluated and those with low binding energies and appropriate poses were shortlisted.

### 2.4. Representation of the biomolecular interactions

The intermolecular interactions were obtained using LigPlot+ [48] and interactively visualized using PyMOL [31].

### 2.5. Evaluating the anthelmintic propensity of compounds

The anthelmintic propensity was computed using the prediction of activity spectra for substances (PASS) [49] and the search for anthelmintic related structures was performed *via* DrugBank [50].

### 2.6. Pharmacological profiling

The selected top hits, as well as the known inhibitors, were subjected to *in silico* pharmacological profiling *via* SwissADME [51] and DataWarrior [52]. SwissADME was used to predict the pharmacokinetic and physicochemical profiles, whereas DataWarrior was used to predict toxicity [52]. The parameters predicted included donors and acceptors of hydrogen bonds, molecular weights, absorption in the intestines, Cytochrome P450 inhibition, permeability glycoprotein substrate, tumorigenicity, mutagenicity, irritancy, and reproductive effects.

### 2.7. Molecular dynamics and MM-PBSA calculations

GROMACS v2018 [32] was used for the molecular dynamics (MDs) of the complexes. The *Na*-GST-3 topology was generated in GROMACS using the GROMOS96 43a1, and PRODRG [53] was used for generating topologies of ligands set at EM: No; Charges: Full; and Chirality: Yes. The generated topologies were merged with that of the protein to form the complexes. The production simulations were ran for 100 ns and the free binding energies were computed using g_mmpbsa [29] utilizing frames in steps of 1 ns.

### 2.8. Quality assessment of leads

The ligand evaluating metrics used were fit quality (FQ), ligand efficiency (LE), ligand efficiency dependent lipophilicity (LELP) and ligand efficiency scale (LE_Scale). These metrics were computed using equations (1)–(4) [54–56].

(1) $$LE=\frac{-BE}{NHA}$$

(2) $$LE_{-}Scale\;=0.873\times e^{\left(-0.026\times NHA\right)}-0.064$$

(3) $$FQ=\frac{LE}{LE_{-}Scale}$$

(4) $$LELP=\frac{\log P}{LE}$$

where BE = binding energy, NHA = number of non-hydrogen atoms and logP = lipophilicity.

## 3. Results and discussions

### 3.1. Protein structure retrieval and molecular dynamics simulation

**Fig. 1.**
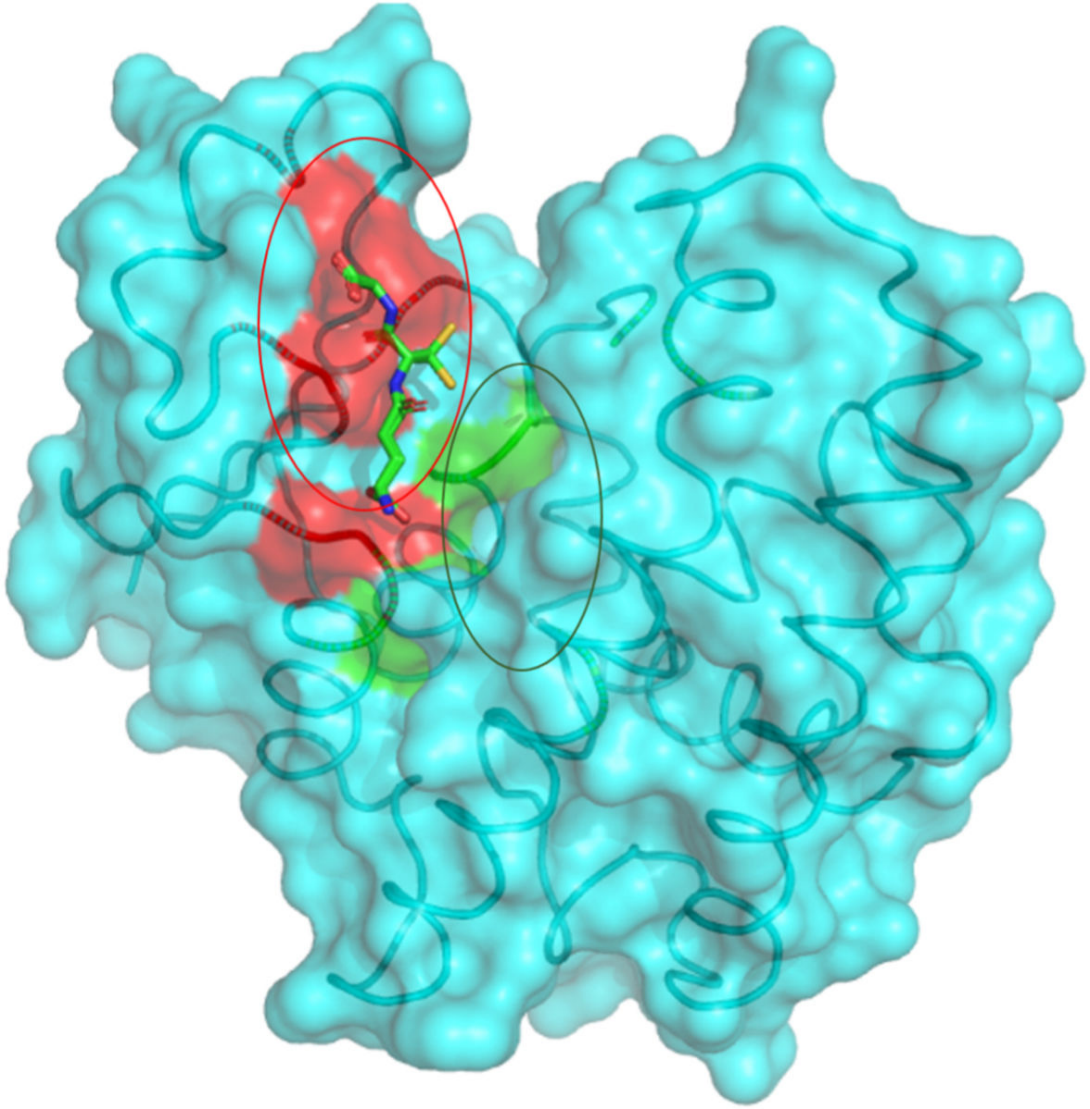
A transparent surface representation of the crystallographic monomeric *Na*-GST-3. The binding site circled in yellow and colored red indicates the glutathione binding G-site, whereas the binding site circled in blue and colored green indicates the ligand-binding H-site. GSH is represented as sticks.

The X-ray structure of *Na*-GST-3 with PDB ID: 3W8S [19] solved at a resolution of 2.07 Å was used for structural analysis. The *Na*-GST-3 monomer has a ligand-binding H-site as well as glutathione binding G-site [19]. The G-site residues are identical to those in other organisms since the G-site is highly conserved [19]. However, the ligand-binding H-site is responsible for the varying substrate specificity across organisms [19]. A surface representation of the structure was generated using PyMOL [31] and it shows the ligand-binding H-site and glutathione bound to the G-site (Fig. 1).

**Fig. 2.**
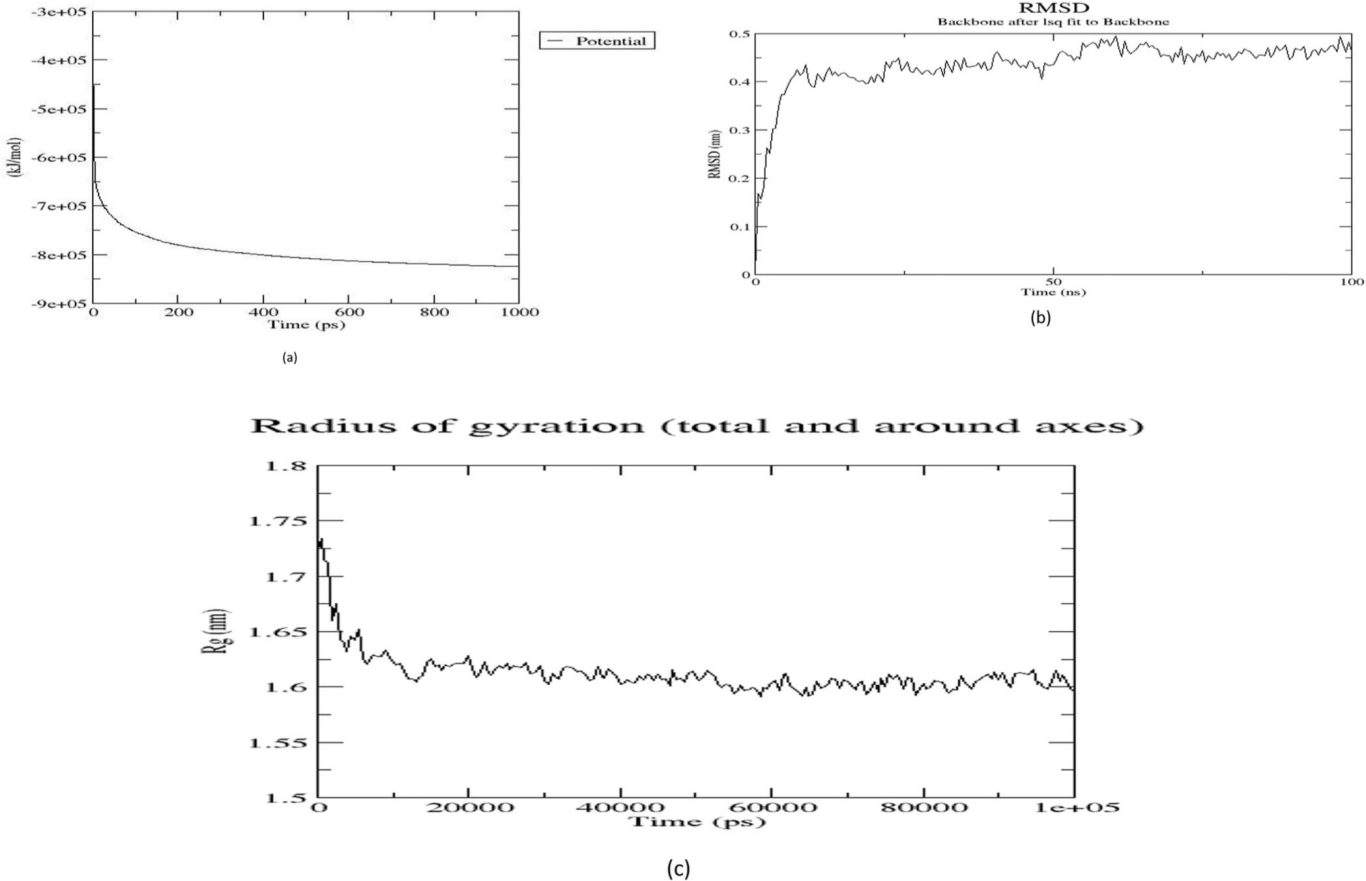
Molecular dynamics simulation of *Na*-GST-3 structure. (a) A plot of potential energy (kJ/mol) against time (ps). The plots decreased exponentially during the initial phases and converged with a potential energy of −8.28e+05 kJ/mol from 800 ps to 1000 ps. (b) A graph showing the RMSD of backbone atoms (nm) against time (ns). The RMSD stabilized between 0.38 nm and 0.5 nm. (c) A plot showing the radius of gyration (nm) versus time (ps). This graph represents the protein structure compactness. A stable plot was observed around 0.16 nm.

MD is a computational technique employed in predicting the time-dependent behavior of the molecular system [57]. Minimization of the *Na*-GST-3 protein structure before MD ran reduced potential energy and eliminated inappropriate geometry [58] with the maximum force less than the allowable tolerance set before the energy minimization step. Initial potential energy (E_pot_) of approximately −4.5 × 10^5^ kJ/mol was observed to decrease exponentially and converged around – 8.3 × 10^5^ kJ/mol (Fig. 2a). E_pot_ value of −8.2 × 10^5^ kJ/mol was obtained after minimization for 1000 ps. Moreover, the maximum force converged to 9.5 × 10^2^ kJ/mol, which was lower than the allowable tolerance of 1000 kJ/mol, denoting a successful energy minimization step.

Furthermore, the protein stability during the MD simulation was evaluated by computing the RMSD, which accounted for the deviations of the protein atoms and the protein backbone [59]. The plot of the RMSD (Fig. 2b) showed a steep increase from 0.00 to 0.41 nm within the first 6 ns. It then fluctuated between 0.38 nm and 0.50 nm averaging at 0.44 nm. Furthermore, radius of gyration (Rg), a means of calculating the compactness of the structure and atom distribution of atoms to its centroid was computed [60]. The radius of gyration decreased sharply from 0.1725 nm to around 0.16 nm during the first 10000 ps (Fig. 2c) and then stabilized over the next 90000 ps of the simulation. The steady Rg was observed (Fig. 2c) was indicative of a stably folded protein [61].

### 3.2. Validation of docking

**Fig. 3.**
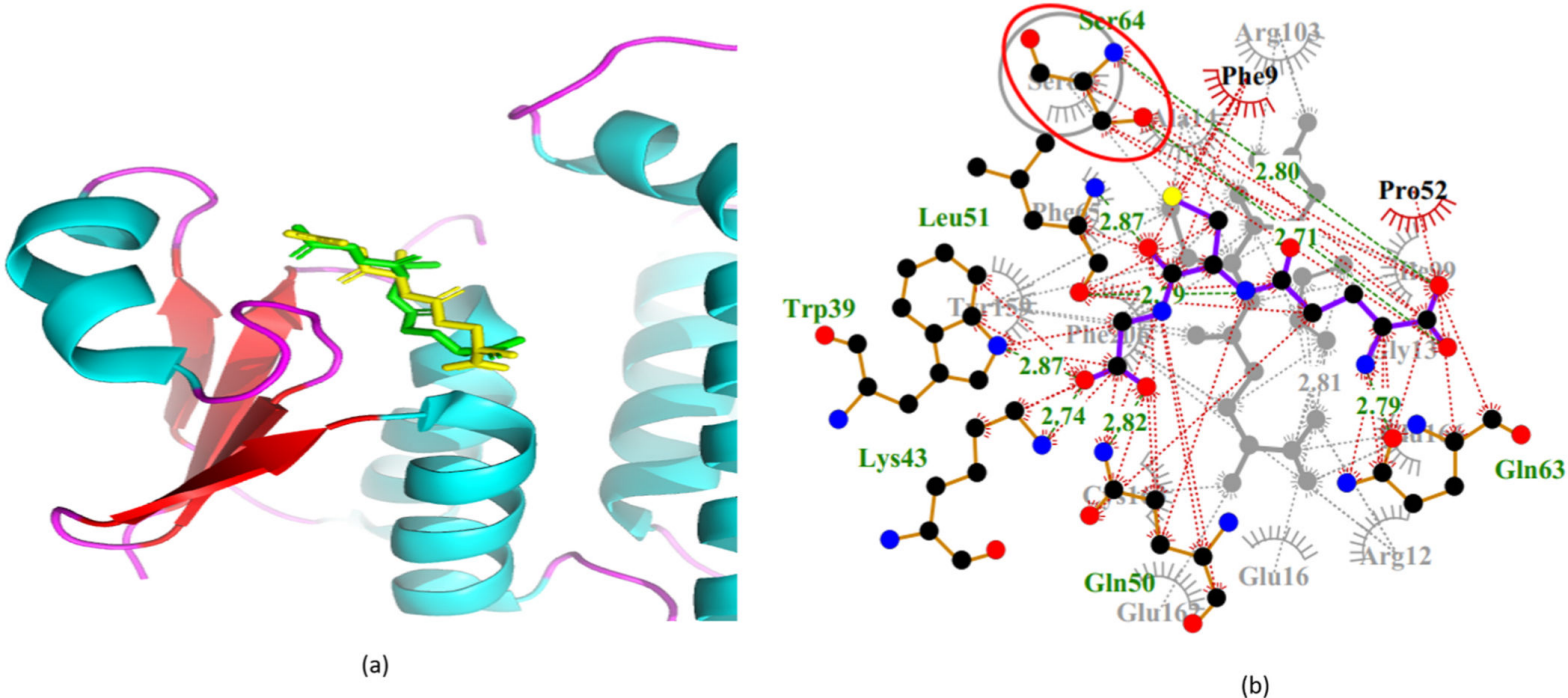
(a) The cartoon represents the superimposition of the re-docked glutathione against the co-crystal ligand. The re-docked glutathione is represented as a green stick whereas the co-crystallized glutathione is represented as a yellow stick. (b) Superimposed LigPlot + representation of overlapping residues between co-crystallized and re-docked glutathione (GSH) ligand. Overlapping residue Ser64 is circled red.

The capability of AutoDock Vina to accurately reproduce the docking poses and differentiate between inactive and active compounds was evaluated. The redocked GSH and crystallographic GSH were superimposed against each other (Fig. 3a). An RMSD of 1.993 Å was computed *via* LigAlign [36], as a measure of the alignment between re-docked GSH and the crystallographic GSH (PDB ID: 3W8S). An RMSD value of <2.0 Å was deemed acceptable [62]. The computed RMSD is redolent of AutoDock Vina’s ability to reproduce a similar pose of GSH. Further analyses of the mechanism of binding using LigPlot + revealed an overlap of the critical amino acid residue Ser64, between the co-crystallized and re-docked complexes (Fig. 3b).

The ROC curve is used to evaluate the capability of the AutoDock protocol to classify active from inactive compounds [63]. The AUC is a plausible metric in evaluating the docking protocol’s potential in classifying docked active and inactive compounds. An AUC value of 1 indicates a perfect categorization whereas an AUC lower than 0.5, describes a poor or worthless categorization between decoys and active ligands [37].

The five inhibitors used to develop the ROC are Cibacron blue, chenodeoxycholic acid, alizarin, lithocholic acid, and ellagic acid. A basic local alignment search tool (BLAST) [64] revealed *Heligmosomoides polygyrus* GST had a 54.37% sequence identity with *Na*-GST-3. *Heligmosomoides polygyrus* GST also shared similar binding site residues (H-site) with *Na*-GST-3 including Gly13 and Ala14 at the α domain as well as Tyr95 and Phe206 at the α_β_ domain [65]. Ellagic acid, alizarin, and chenodeoxycholic acid, on the other hand, were tested against *Echinococcus granulosus* (EgGST2–3) which had a 32.55% sequence identity with *Na*-GST-3, and their respective IC_50_ values were 12 ± 0.6 μM, 52 ± 6 μM, and 53 ± 3 μM [38]. The AUC values generated for the five active ligands with 225 decoys screened against *Na*-GST-3, *Na*-GST-2, and *Na*-GST-1 receptor were 0.731, 0.709, and 0.681, respectively (Supplementary Fig. 1), indicating reasonably good discrimination [66,67].

### 3.3. Library pre-filtering and docking

A total of 2500 out of 3182 compounds were obtained after pre-filtering. This was after eliminating compounds with undesirable functional groups [46,68]. The remaining 2500 compounds were virtually screened against the *Na*-GST-3 ligand binding H-site composed of essential residues Gly13, Ala14, Phe/Leu65, Tyr95, Phe106, Phe206, Glu162, and Arg120 [19]. The top 5% totaling 125 hits were selected based on high binding affinity. Shortlisting the top 5% increased the likelihood of the true positives in the final selection. All the hits that were excluded failed to dock deeply within the binding pocket. A total of 19 compounds were chosen depending on the poses and binding energies. A range of −10.8 to −9.3 kcal/mol (Table 1) were obtained for the compounds. The compound with the highest binding affinity was vobtusine with binding energy of −10.8 kcal/mol. Moreover, the binding affinities of five standard inhibitors used in the generation of the ROC curve were juxtaposed to the top 19 hits. All the known inhibitors except Cibacron blue had lower binding affinities, relative to the 19 hits (Table 1). However, the Cibacron blue was observed not to dock deep in the pocket (Supplementary Fig. 2).

### 3.4. Prediction of the protein-ligand interactions

**Fig. 4.**
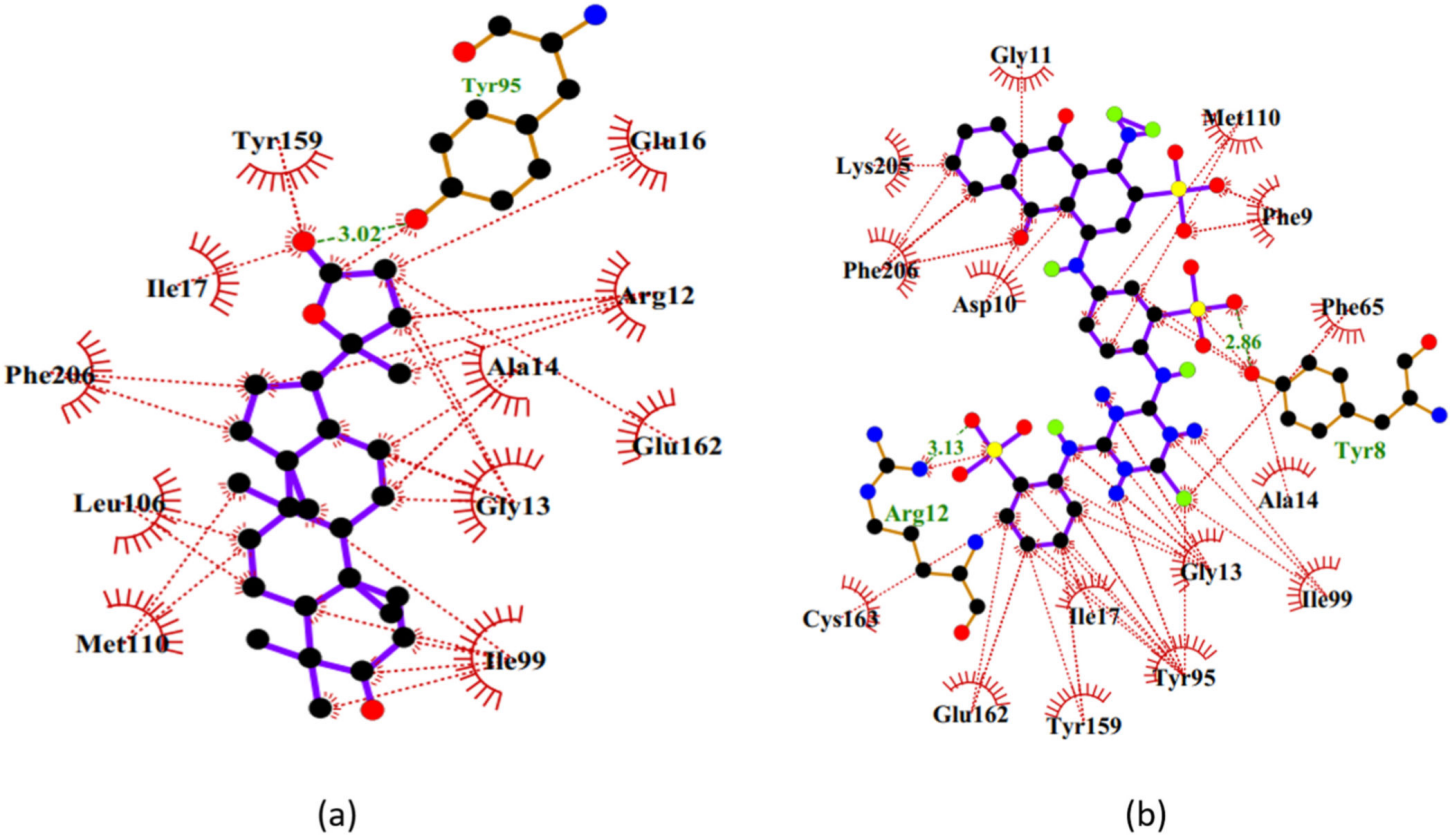
The 2D protein-ligand interactions of (a) Dammarane triterpene13 and (b) Cibacron Blue. The inhibitor**s** are represented as blue sticks.

The binding mechanisms of the top 19 compounds were elucidated using the hydrophobic and hydrogen bonds, which are essential for ligand stability. The structures of the ligands as well as the different functional groups and key active site residues are essential for the protein-ligand complexes [69]. The number bonds and lengths formed with the experimentally determined residues were obtained (Table 2). Thirteen compounds formed intermolecular hydrogen bonds with the residues, and the strong bonds are those with short lengths [70]. The protein-ligand interactions generated for Dammarane Triterpene13 and Cibacron Blue complexes are shown in Fig. 4. Dammarane Triterpene13 formed hydrophobic contacts with Gly13, Ala14, Glu162, Phe206, and Tyr95, and hydrogen bonding of length 3.02 Å with Tyr95. Cibacron had two hydrogen bond interactions with Tyr8 at length 2.86 Å and Arg12 at 3.13 Å, and eight hydrophobic bonds with Tyr8, Phe9, Gly13, Ala14, Phe65, Tyr95, Glu162, and Phe206. The shortest bond length of 2.46 Å was between 3′-*O*-beta-D-glucocalotropin and Tyr95 (Table 2). ZINC85999636 with a binding energy of −9.3 kcal/mol had two hydrogen bonds with Gly13 and Tyr95 at lengths 3.03 Å and 2.27 Å, respectively. It also formed hydrophobic interactions comprising Gly13, Ala14, Ser64, Phe65, Phe206, and Tyr95. Other ligands namely ZINC34518176 and ZINC14825190 were stabilized by hydrophobic interactions with Gly13, Ala14, Tyr95, and Glu162. Moreover, 4-*O*-(4^”^-*O*-galloyl-alpha-L-rhamnopyranosyl) ellagic acid with a binding affinity of −9.4 kcal/mol had the highest number of five hydrogen bonds. This compound formed one hydrogen bond separately with each of Leu51, Tyr8, and Arg12, as well as two hydrogen bonds with Gln50 (Table 2). Overall, the hits formed intermolecular hydrogen and hydrophobic bonds with most of the experimentally reported residues in the H-site including Phe/Leu65, Phe106, Phe206, Glu162, and Arg120, with Gly13, Ala14 and Tyr95 considered critical [19]. Residues namely Phe9, Ser64, Gln50 and Leu51 which formed interactions with the crystallographic GSH were found to interact with some of the 19 hits (Table 2). However, Lys43, Gln50, Gln63 and Pro52 did not interact with any of the 19 hits.

### 3.5. Exploring the hits for anthelmintic related biological activities

The top 19 hits were further assessed for their anthelmintic-related biological activity *via* PASS [49], which employs Bayesian models for biological activity prediction of a compound. The training set for PASS Bayesian model was built on the known biological activities of over 26, 000 compounds [49]. PASS compares the chemical structure of the queried with those in the database to predict their biological activity. Anthelmintics are a broad stream of both synthetic and natural-based products used to treat helminth infections [71]. PASS was employed in previous studies [72,73], and the anthelmintic activity was experimentally corroborated, reinforcing the use of PASS for predicting the anthelmintic tendency of compounds. A total of 9 out of 19 hits were predicted to have anthelmintic activity with probability of activity (Pa) > probability of inactivity (Pi) (Supplementary Table 1). Therefore, it is expedient to test the compounds *via in vitro* assaying to corroborate the pharmacology of the compounds [74].

ZINC85999636 was predicted to have Pa and Pi of 0.540 and 0.005, respectively (Supplementary Table 1). Moreover, structural similarity searches *via* DrugBank [50] and literature sources were employed to support the predictions. DrugBank search revealed that ZINC85999636 had a structural similarity of 0.573 to Hesperidin. Hesperidin has a wide range of pharmacological effects, including anthelmintic biological activity. It was reported to be effective in the inhibition of important *Schistosoma mansoni* enzymes [75].

ZINC14825190 had Pa of 0.486 and Pi of 0.025 (Supplementary Table 1), and structural analysis revealed it contains a chromen-6-one substructure. A structural similarity search using DrugBank retrieved genistein with a score of 0.625. Studies have shown that genistein possesses significant deworming properties [76]. Genistein was identified to be a key anthelmintic component of some plant species including *Flemingia vestita* [76]. Genistein induces estrogenic effects by binding to the estrogen receptors of *Echinococcus multilocularis* and *Echinococcus granulus* metacestode [77]. Furthermore, genistein has been demonstrated to be potent against helminths *Fasciola hepatica, Fasciolopsis buski* [78], and *Raillietina echinobothrida* [79,80]. It inhibits the enzymes responsible for glycolysis and glycogenolysis in *Raillietina echinobothrida* [79,80].

ZINC28462577 had a Pa of 0.457 and DrugBank structure similarity of 0.925 with diosmetin. Diosmetin is a flavone that possesses substantial anthelmintic activity. *Digtaria insularis* showed an anthelmintic effect on nematodes possibly due to the flavones including diosmetin constituent [81]. Additionally, structural analysis of ZINC28462577 revealed a 2-phenylchromen-4-one substructure, which is associated with anthelmintic activity [82].

4-*O*- (4”-*O*-galloyl-alpha-L-rhamnopyranosyl) ellagic acid structurally similarity to diosmetin with a score of 0.668. Moreover, it is an ellagic acid derivative. Ellagic acid was shown to have an IC_50_ of 7.3 μM against the helminth *Echinococcus granulosus* GST-1 [38].

17alpha-hydroxycabralealactone is structurally similar to Bevirimat with a score of 0.722, a betulinic acid-like compound [83]. Betulinic acid has been reported to be the anthelmintic component of *Berlina grandi* florastem bark extract [84]. This was as a result of the reactive carbonyl and the hydroxyl moiety [82].

ZINC13411589 is structurally similar to 1-(3-methylphenyl)-1H-benzimidazol-5-amine with a score of 0.53. 1-(3-methylphenyl)-1H-benzimidazol-5-amine belongs to a family of compounds known as phenylbenzimdazoles.

ZINC95486378 also had 0.625 structural similarity with Niclosamide, a known and safe anthelmintic drug that has been tested *in vivo* with no side effects [85]. It was reported to cause tegumental changes in *Haplorchis taichui* [86].

ZINC34518176 had no PASS predicted anthelmintic activity. Nonetheless, it was structurally similarity to Terpine-4-ol with a score of 0.71. Terpine-4-ol possesses remarkable ovicidal and larvicidal properties against *Haemonchus contortus* [87]. When the concentration is at 3.5 mg/ml, there is 100% inhibition to the hatching of eggs by *Haemonchus contortus.* It was also efficacious against *Haemonchus contortus* larvae causing 85.7% and 82.4% inhibition at concentrations of 56 mg/ml and 3.5 mg/ml, respectively [87].

### 3.6. Drug-likeness prediction studies

The drug-likeness of the top 19 hits (Table 3) was assessed using the Lipinski’s Rule of Five (RO5) [88]. The physicochemical parameters considered include a logarithm of *n*-octanol/water partition (logP) ≤ 5; molecular weight (MW) ≤ 500 Da; hydrogen bond acceptors (HBA) ≤ 10; and hydrogen bond donors (HBD) ≤ 5. The octanol-water partition coefficient or lipophilicity is important to let solutes (drugs) move through the interfacial region of cell membranes. Hydrogen bond acceptors and donors are also important information of hydrogen bonds which are critical in ligand stabilization [89]. Moreover, the molecular weight affects the absorption of a drug into the blood circulation [90]. Per the RO5, a drug-like compound must not violate more than two criteria [88]. Sixteen compounds were predicted to be drug-like per RO5 compliance using SwissADME [51]. Of the known GST inhibitors, only Cibacron blue violated two of the RO5, consisting of a molecular weight of 771.13 g/mol, which was above the allowable 500 g/mol and 14 hydrogen bond acceptors, which was 4 more than the allowable 10. The remaining 4 inhibitors were all predicted to be RO5 compliant (Table 3).

### 3.7. Pharmacokinetics and toxicity profile predictions of selected hits

Key pharmacological profiles comprising permeability glycoprotein (P-gp) substrate, gastrointestinal absorption, inhibition of Cytochrome P450 3A4 (CYP3A4), mutagenicity, tumorigenicity, reproductive effect, and irritancy were investigated in this study.

Human intestinal absorption (HIA) is a key pharmacokinetic property [91]. SwissADME utilized a BOILED-Egg model to predict gastrointestinal absorption [51]. The higher the gastrointestinal absorption, the better the bioavailability of the compound. A total of 11 out of 19 hits had high intestinal absorption (Supplementary Table 2). Cibacron blue was the only standard inhibitor predicted to have low gastrointestinal absorption (Supplementary Table 2).

Furthermore, P-gp is a significant ATP-dependent membrane protein responsible for the pumping of intracellular foreign materials to the extracellular spaces of cells [92]. It has broad specificity and also plays an important function in safeguarding the central nervous system from xenobiotics [51]. Moreover, a study has implicated P-gp to be a contributing factor to anthelmintic resistance [92]. Resistance to anthelmintics can be caused by the concentration of the drug at the target site being altered by a Pgp-mediated interaction. This mechanism has been demonstrated in human cells [92]. Out of the twenty hits considered, only two compounds namely vobtusine and 3′-*O*-beta-D-glucocalotropin were predicted as P-gp substrates (Supplementary Table 2).

The Cytochrome P450 3A4 (CYP3A4) is a major drug metabolizing enzyme [93]. CYP3A4 inhibition may lead to pharmacokinetics-related drug-drug interactions and alter the response of some drugs [93]. Two hits namely ZINC00134782 and ZINC85999636 were predicted to be CYP3A4 inhibitors (Supplementary Table 2). SwissADME utilizes a support vector machine algorithm (SVM) to predict the pharmacological profiles [51].

DataWarrior was employed in predicting the propensity of the molecules to cause an undesirable effect in the entire organism or an organ [94]. Drugs have been withdrawn in clinical trials due to toxicity issues, hence prior knowledge of safety is important [95]. DataWarrior predicted the likely mutagenic, tumorigenic, reproductive effect and irritancy of the selected compounds [52]. Amongst the hits, only ZINC95486378 was predicted to be mutagenic, tumorigenic, have a reproductive effect, and an irritant. Cibacron blue was also predicted to be toxic per all the toxicity parameters considered (Supplementary Table 2).

### 3.8. Potential lead compound selection

**Fig. 5.**
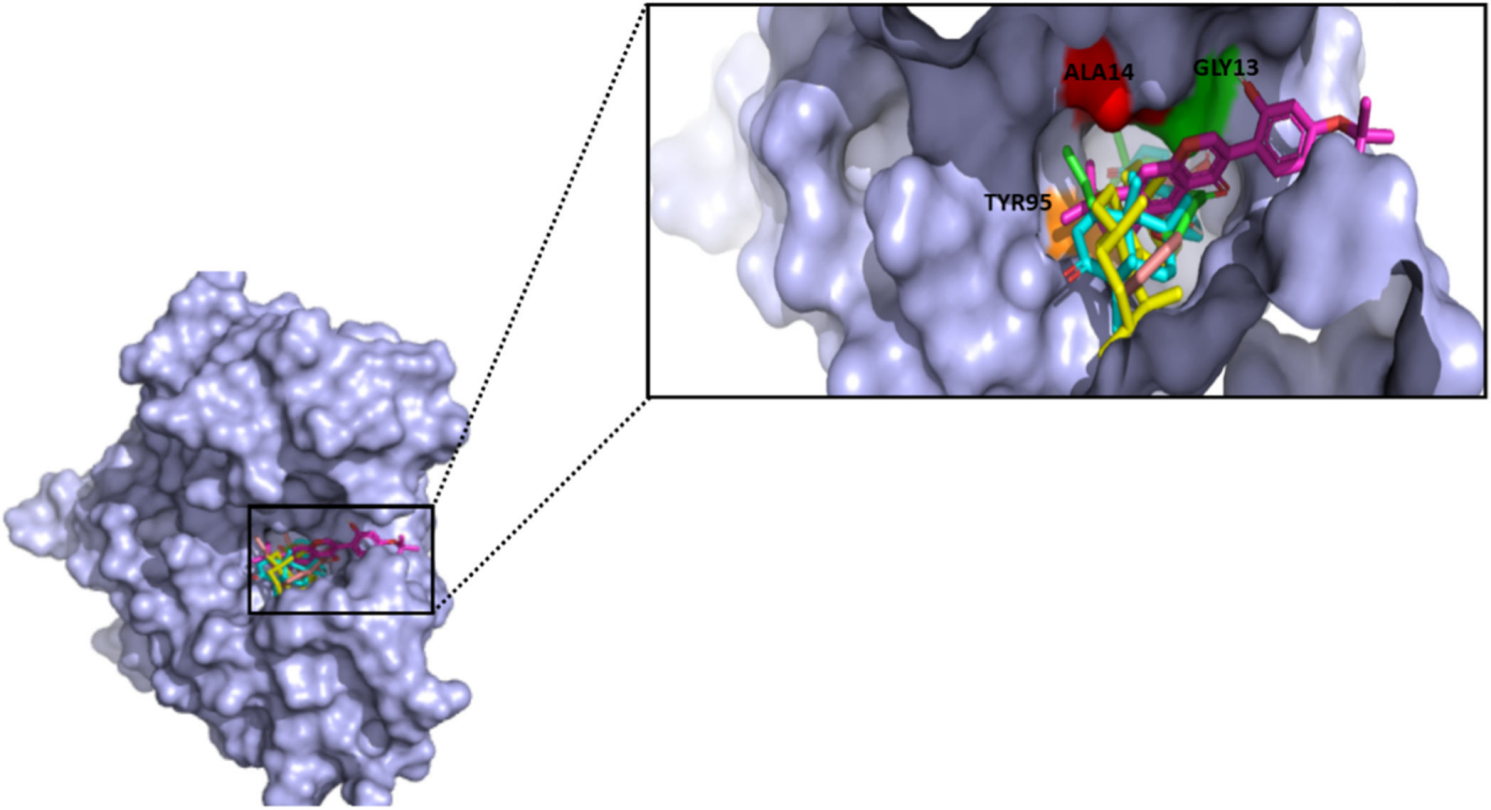
The complex structure showing three of the putative leads docked deep inside the pocket of *Na*-GST-3. They also interacted with the critical residues (Gly13, Ala14, and Tyr95). The surface representation of the protein is colored light blue, whereas the ligands are rendered as sticks. Dammarane triterpene13, ZINC35418176, ZINC85999636, and ZINC14825190 are shown as cyan, yellow, brown, and magenta sticks, respectively.

A lead is a molecule that could potentially be developed into a therapeutic molecule by optimizing its features [88]. Considering the binding energies, protein-ligand interactions, biological activity predictions, anthelmintic structural similarity searches *via* DrugBank, and the pharmacological profiles, four compounds were selected as putative leads with the potential to inhibit *Na*-GST-3. The selected compounds were ZINC85999636, Dammarane triterpene13, ZINC14825190, and ZINC35418176 (Table 4). The four promising leads docked deep inside the binding pocket of *Na*-GST-3 (Fig. 5) with binding energies lower than −9.0 kcal/mol (Table 5). Their mechanisms of binding showed that ZINC85999636 formed hydrogen bonding with critical residues Tyr95 and Gly13. Also, Dammarane triterpene13 formed a hydrogen bond with Tyr95. Both Dammarane triterpene13 and ZINC85999636 formed 11 hydrophobic contacts (Table 2). ZINC35418176 and ZINC14825190 separately formed nine hydrophobic contacts with the critical binding site residues Gly13, Ala14, and Tyr95, which are reported to play major roles in ligand binding [19]. Furthermore, all four compounds had related anthelmintic activity. ZINC85999636 was predicted to have the highest probable anthelmintic activity of 0.540. It is structurally similar to Hesperidin, which is known to have anthelmintic properties [96]. ZINC14825190 had a structural similarity of 0.625 to genistein. From the aforementioned, genistein is an efficacious anthelmintic with wide-ranging anthelmintic activity against helminths *Fasciola hepatica, Fasciolopsis busk* [78], and *Raillietina echinobothrida* [79]. Additionally, ZINC35418176 which is structurally similar to terpine-4-ol with a score of 0.71, had significant anthelmintic activities against helminth *Haemonchus contortus* [87]. Dammarane Triterpene13 was predicted to possess anthelminthic activity. Other hit compounds such as Olibanumol and ZINC95486223 had a good druglike outlook including low binding energy, good pharmacokinetic and physicochemical properties. However, Olibanumol and ZINC95486223 may be unique scaffolds.

### 3.9. Screening of selected compounds against Na-GST-2 and Na-GST-1

The four selected compounds and the five known helminth GST compounds were screened against the *Na*-GST-1 and *Na*-GST-2. This was done to probe into the individual binding affinity of the selected compounds against other *Na*-GSTs, which also perform the heme detoxification function as *Na*-GST-3 [19,20,22]. The protein-ligand interaction analysis *via* LigPlot+ was performed to establish the possibility of a similar mechanism of binding among the selected compounds.

*Na*-GST-1 and *Na*-GST-2 are homologues of *Na*-GST-3 with sequence identities of 59.2% and 75.7% to *Na*-GST-3, respectively [19]. Also, they share a number of hydrophobic residues in the ligand-binding site (H-site). The α3 domain contains Gly13, Ala/Leu14 and Leu/Phe65, whilst the α/β domain contains Tyr95, Phe/Tyr106 and Phe206 at [20].

The docking results showed that Dammarane triterpene13, ZINC14825190, and ZINC85999636 had appreciably high binding affinities against all the three *Na*-GSTs, with ZINC14825190 having the highest binding affinity of −10.8 kcal/mol against *Na*-GST-2 (Table 5). Amongst the standard inhibitors used, Cibacron blue had a relatively high binding affinity of −9.0 kcal/mol for *Na*-GST-1 and −9.4 kcal/mol for *Na*-GST-2. All compounds had high binding affinities except ZINC35418176 which had relatively low affinities of −6.1 kcal/mol for *Na*-GST-1 and −6.6 kcal/mol for *Na*-GST-2 (Table 5).

**Fig. 6.**
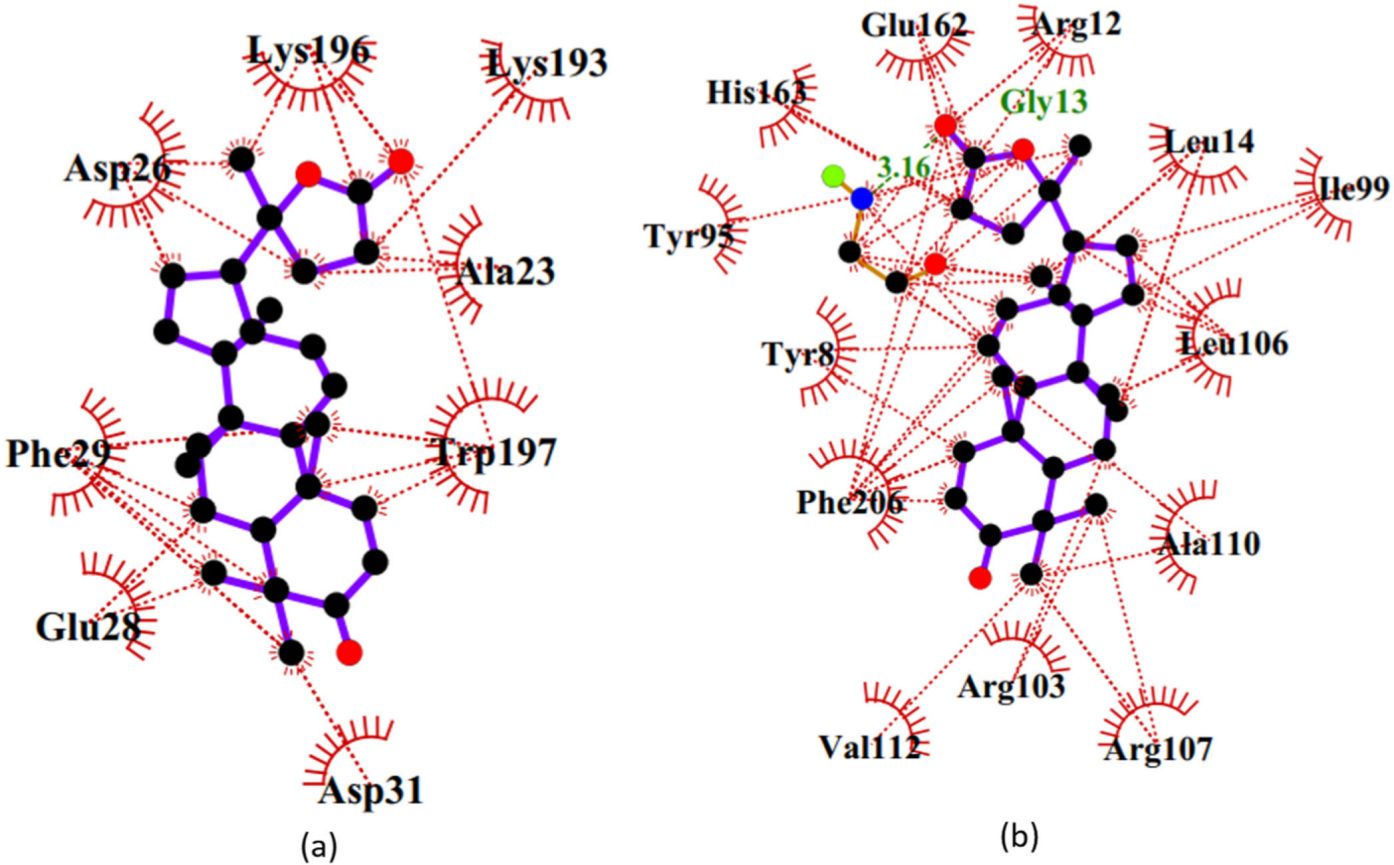
2D protein-ligand representation of Dammarane triterpene13 binding to (a) *Na*-GST-1, and (b) *Na*-GST-2.

Additionally, profiling of protein-ligand interactions showed similar interacting residues including critical residues Tyr95, Ala/Ile14, and Ile/Arg99 (Figs. 4a and 6) among the three receptors. This finding corroborated the *Na*-GSTs ligand-binding mechanism similarities reported elsewhere [19].

### 3.10. Molecular dynamics simulations of complexes

#### 3.10.1. Molecular dynamics

**Fig. 7.**
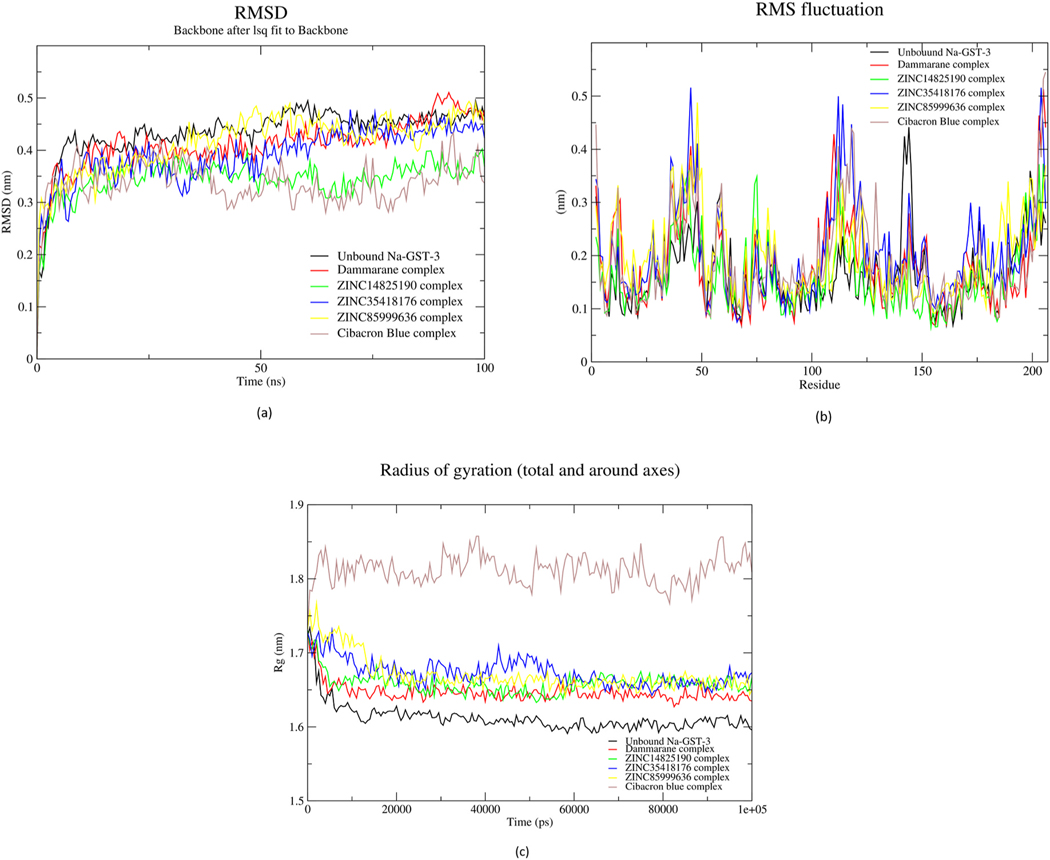
The RMSD (a), RMSF (b) and Rg (C) plots of the complexes and unbound protein against time in ps. In all three graphs, black, green, blue, and yellow represent the protein core, *Na*-GST-3-Dammarane Triterpene13, *Na*-GST-3-ZINC14825190, *Na*-GST-3-ZINC35418176, *Na*-GST-3-ZINC85999636 and *Na*-GST-3-Cibacron blue complexes, respectively.

A 100 ns MD simulation of protein-ligand complexes of ZINC35418176, ZINC85999636, ZINC14825190, Dammarane triterpene13, and known inhibitor Cibacron blue were performed to investigate the conformational and structural dynamics [97]. An acceptable measurement of this stability is the root-mean-square deviation (RMSD) [98]. The deviations undergone by the backbone atomic coordinates during simulations are evaluated using the RMSD [98]. A plot of the RMSD over time (ns) showed all the fluctuations of the four potential lead complexes ascended from 0 nm and stabilized around the same range of 0.4–0.45 nm. *Na*-GST-3-Dammarane triterpene13 complex attained stability at the beginning of the simulation at approximately 0.4 nm but eventually spiked up around 80 ns (Fig. 7a). Despite the spike, it had similar fluctuations as the protein core with an RMSD closer to it. On the other hand, the *Na*-GST-3-ZINC85999636 complex fluctuated between 0.4 nm and 0.45 nm, and the *Na*-GST-3--ZINC35418176 complex attained stability after approximately 75 ns, even though, it had an extreme deviation from the unbound protein. Fluctuations began to stabilize between 0.4 nm and 0.45 nm. The *Na*-GST-3-Cibacron blue complex also rose from 0 nm but deviated from the fluctuations of the protein core. It stabilized between 0.3 nm and 0.4 nm, indicating not much change from the initial protein backbone coordinates relative to the other three complexes.

The root-mean-square fluctuation (RMSF) was also used as a metric to evaluate the flexibility of the protein residues [99,100]. The fluctuations of the individual residues contributing to the protein-ligand complex over the 100 ns simulation were analyzed. All five complexes including Cibacron blue possessed similar fluctuations as the protein core with little deviations. High fluctuation levels were observed at residue positions 30–50 and 100–125 (Fig. 7b).

Finally, the protein compactness was evaluated using the radius of gyration (Rg) [101]. A stably folded complex would maintain a steady radius of gyration. All the four lead complexes deviated from the unbound protein by at least 0.05 nm (Fig. 7c). Dammarane triterpene13 complex stabilized around 1.65 nm, ZINC35418176 complex had fluctuations which also stabilized at 1.65 nm but only after the 60000^th^ ps and ZINC85999636 stabilized between 1.65 nm and 1.655 nm. These observations revealed that the structures of Dammarane triterpene13 and ZINC85999636 complexes were compact after simulation. Cibacron blue, on the other hand, deviated much from the protein core with its fluctuations stabilizing between 1.8 nm and 1.85 nm.

#### 3.10.2. Binding mode analysis

The protein-ligand interactions of the five complexes were mapped post molecular dynamics simulation. A superposition of the pre-MD and post-MD binding modes revealed common interacting residues namely Gly13, Ala14, Ile99 and Phe206 for ZINC14825190 (Supplementary Fig. 3). Residues comprising Gly13, Ala14, Ile99, Arg103, Leu106, Glu 162, and Cys163 interacted with both binding modes of ZINC35418176. Additionally, dammarane triterpene13 interacted with Ile99, Glu16, Tyr159, Ile17, Ala14, Tyr159, Leu106 and Phe206. Overall, Gly13 and Ala14 were shown to interact with all the complexes after analyzing their pre- and post-MD binding poses.

#### 3.10.3. Estimation of the free binding energy using the MM-PBSA method

**Fig. 8.**
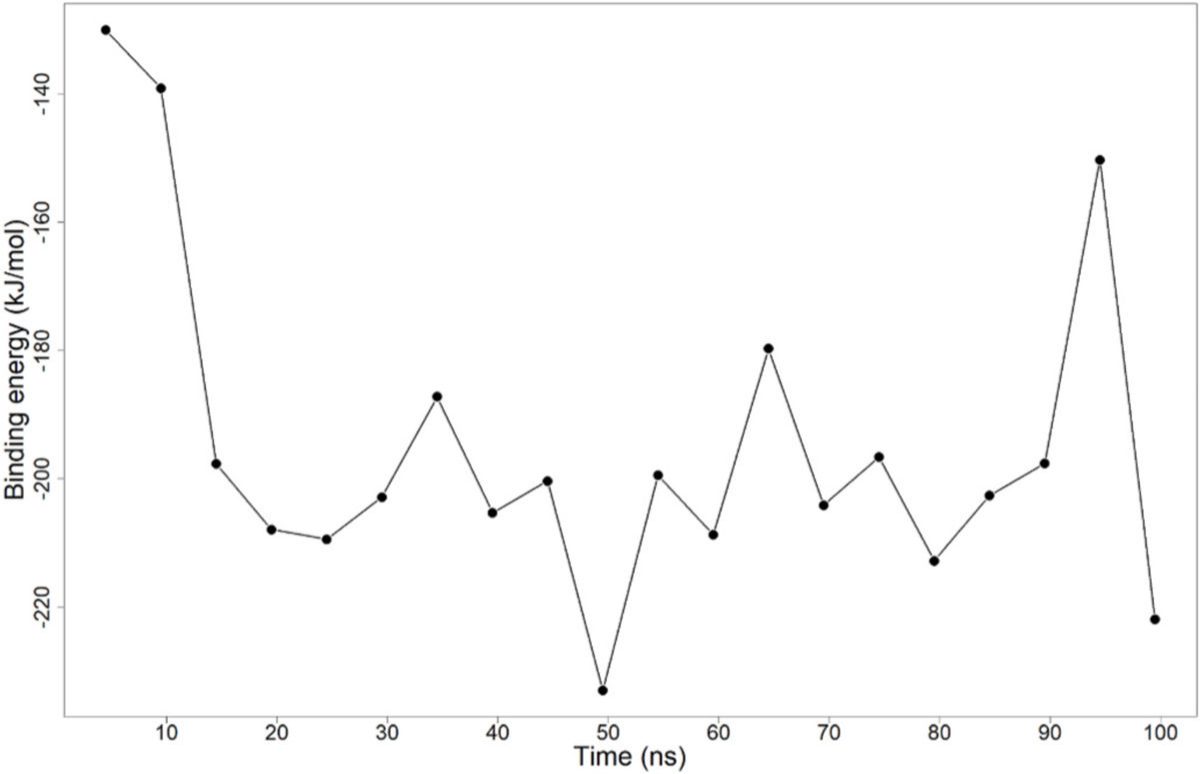
The binding free energy graph of Dammarane Triterpene13 in kJ/mol against time ns.

The MM-PBSA was employed to estimate the binding free energies of the complexes [102], which addresses the flaws of existing scoring functions including the handling of solvent effects [97] and numerous approximations in the scoring functions [103]. The MM-PBSA method estimates the van der Waals, polar solvation, non-polar solvation, and electrostatic energies, contributing jointly to the free binding energies. The average binding energy was calculated for all five compounds. The average binding free energy of Dammarane Triterpene13 was −194.34 kJ/mol (Table 6) and it dropped steeply from −150 kJ/mol to −220 kJ/mol over the first 30 ns. It then appeared to be undulating over the next 70 ns, with a nadir of around −230 kJ/mol at 60 ns (Fig. 8). Also, the free binding energy of ZINC85999636 averaged at −187.99 kJ/mol (Table 6), with an initial value of −176.843 kJ/mol (Supplementary Fig. 4a). The plot peaked at 35 ns with energy of 48.129 kJ/mol and then undulated after the next 10 ns. At the end of the 100 ns, 29.894 kJ/mol was obtained as the final binding free energy. The average energy of ZINC14825190 was −209.585 kJ/mol, with −215.326 kJ/mol as the initial energy. It further undulated and peaked at 85 ns (Supplementary Fig. 4b). The average binding free energy of ZINC35418176 was −181.23 kJ/mol (Table 6). At 20 ns, a peak binding energy obtained was 30.452 kJ/mol (Supplementary Fig. 4c). Cibacron blue had low binding energy initially (<0 kJ/mol) during the first 40 ns but increased over the next 60 ns (Supplementary Fig. 4d). In all instances, electrostatic, van der Waals, and non-polar solvation energies contributed favorably to the binding energies of all the complexes (Table 6). However, the polar solvation energy [104], contributed adversely to the total binding energy. Polar solvation energy was prominent in Cibacron blue (Table 6). This might have contributed to the relatively high binding energy (−5.9 kJ/mol).

**Fig. 9.**
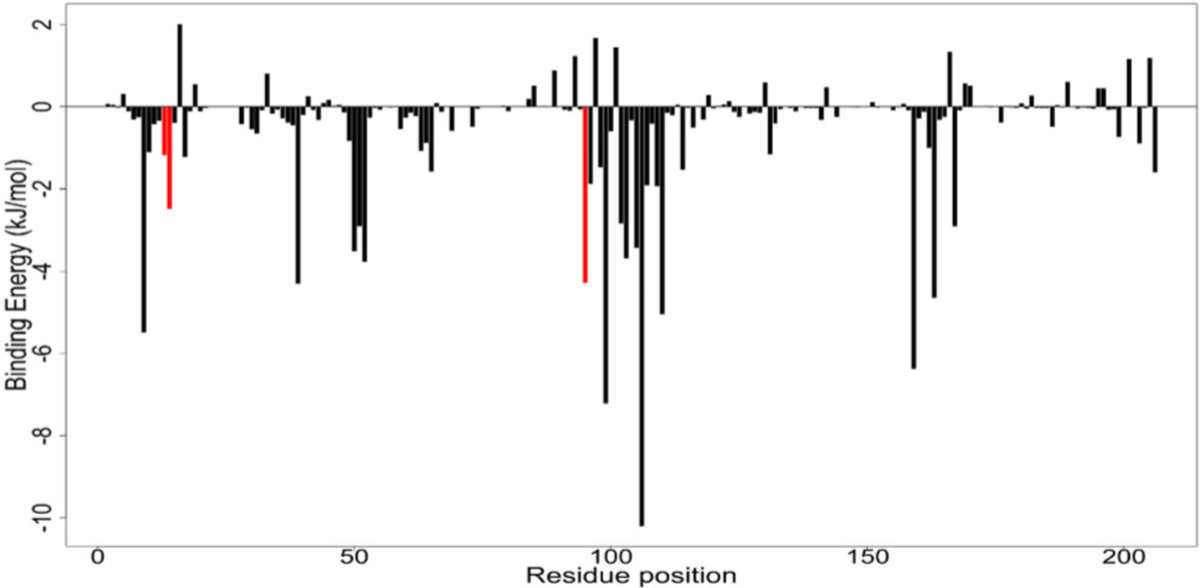
The per-residue energy decomposition of the Dammarane Triterpene13 complex. The suggested critical residues (Gly13, Ala14, and Tyr95) are colored red.

The decomposed energy of each residue was determined. For the four potential leads, the binding contribution of an individual residue ranged from 4.7 kJ/mol to −11.0 kJ/mol, with a number of residues contributing negative energies (Fig. 9, Supplementary Fig. 5). However, the energy contribution plot of Cibacron blue ranged from 55 kJ/mol to −55 kJ/mol (Supplementary Fig. 5d). Critical residue Gly13 contributed −1.2083 kJ/mol, −2.7643 kJ/mol, −2.0865 kJ/mol, −2.3343 kJ/mol and −3.05999 kJ/mol to the binding of Dammarane Triterpene13, ZINC85999636, ZINC35418176, Cibacron Blue and ZINC14825190, respectively (Table 7). Ala14 contributed −2.5027 kJ/mol, −4.5778 kJ/mol, −3.2030 kJ/mol, −0.4831 kJ/mol and −4.4520 kJ/mol to the binding of Dammarane Triterpene13, ZINC85999636, ZINC35418176, Cibacron Blue and ZINC14825190, respectively (Table 7). Tyr95 contributed −4.2508 kJ/mol, −2.4909 kJ/mol, −3.5749 kJ/mol, −2.2440 kJ/mol, and −0.3982 kJ/mol to the binding of Dammarane Triterpene13, ZINC85999636, ZINC35418176, Cibacron Blue and ZINC14825190, respectively (Table 7). Residues Met110 and Cys163 also contributed favorably energies to the binding (Table 7). Therefore, residues Met110 and Cys163 are suggested as critical amino acids for binding.

### 3.11. Quality assessment of selected leads

When selecting leads for optimization in fragment-based drug design (FBDD), a library of lower molecular weight molecules/fragments is screened against a target to identify the low-affinity pharmacophoric elements or potent drug leads needed for high-affinity binding [105]. FBDD aids in the development of useful drug leads with enhanced biological activity [106].

The ligand efficiency (LE) is a plausible metric used in evaluating fragments [106]. LE measures *in vitro* biological activity which pertains to the binding affinity of leads or hits binding to a receptor, based on structural features [55]. The LE of lead-like molecules is at least 0.3 kcal/mol/HA [107]. The selected leads had LE ranging from 0.3097 to 0.475 with ZINC35418176 having the highest LE of 0.475 (Table 8).

Though LE is a useful metric, it fails to account for the different molecular sizes of non-hydrogen atoms, hence, values obtained are not consistent [108]. Therefore, ligands with higher molecular sizes have relatively lower LE than smaller ones. The fit quality (FQ) and ligand efficiency scale (LE Scale) have been proposed as better alternatives and size-independent functions [108,109]. FQ values closer to 1 suggest an ideal binding between the ligands and the receptor [55,56]. All four molecules had FQ values close to 1 (Table 8), which suggests an ideal binding to the receptor. Moreover, the LE Scale of 0.3 or less shows the potential of the molecule [56]. However, all compounds were close to the 0.3 threshold for LE_Scale except ZINC35418176 (Table 8) which had 0.45502.

The Ligand efficiency-dependent lipophilicity (LELP) combines both molecular size and lipophilicity into a single efficiency, addressing shortcomings of other metrics [55,110]. An acceptable range of −10 < LELP <10 is defined for lead-like compounds with −3 < logP <3 [111]. Additionally, compounds in the Lipinski’s zone should have a LELP value less than 16.5 [111]. ZINC35418176 and ZINC14825190 had LELP values that are very close to the threshold for Lipinski’s zone compounds (Table 8). Dammarane Triterpene13 and ZINC85999636 had relatively higher LLP.

Considering these quality assessment metrics, the selected molecules can form essential backbones for the design of novel anthelmintics.

### 3.12. Significance, limitations, and implications of the work

Due to the limited availability of new anthelmintics and the widespread resistance to them, the use of anthelmintics has become more complex. Although, there are various strategies to fight against helminths, the current strategy of treating anthelmintics has challenges. Computational techniques have evolved as a faster and more cost-effective method of finding novel anthelmintic compounds. Even though widely used molecular docking techniques such as AutoDock Vina have shortfalls, robust techniques are used to evaluate their performance to ameliorate the limitations. Several benchmark projects have successfully evaluated their performance [112,113] and herein used AUCs computed from the ROCs. Binding free energies computed from MM-PBSA analysis were used to support the binding affinity of the ligands. Also, the co-crystallized and re-docked ligands were aligned to regenerate the pose as a form of evaluating the molecular docking. The outputs of the validations show that the results of the molecular docking are reasonably acceptable albeit the deficiencies due to sensitivity and specificity. The work further integrates different robust techniques including characterization of binding, prediction of biological activity and mechanisms, structural similarity evaluation, pharmacological profiling, and MD simulations to aid in selecting plausible potential leads. Besides, metrics such as LE, LELP, FQ and LE_Scale were computed to evaluate the efficiency of the ligands. Since the bioactivity of the potential leads was predicted, it needs experimental confirmation. Nevertheless, the predicted results are rich sources of data to support the ongoing search for future hookworm therapeutic molecules. Such an approach prioritizes few molecules for experimentation from large ligand libraries since large-scale *in vitro* screening of compounds is expensive and laborious.

Computational techniques augment the drug discovery processes for the early identification of potential leads with druglike and good pharmacological profiles. This work provided novel insights into the binding mechanisms of *Na*-GSTs and known inhibitors as well as the potential leads, which could lead to the design of novel structures with the potential to possess *Na*-GST-3 activity. The predicted activity provides insights into possible inhibition of the receptor by the compounds. Also, *in silico* techniques have been used to elucidate the binding mechanisms of anthelmintics [114].

*In silico* inferences do not replace experimental results but rather complements them. In the search for COVID-19 drugs, a library of about 1.3 billion compounds was screened against a key receptor of the causative virus SARS-CoV-2. This work freely made available prioritized compounds for biotherapeutic evaluation [115]. Similarly, potential anti-SARS-CoV-2 [116] and anti-Ebola VP24 [117] compounds were predicted from a library of African natural products.

Even though, the publication of experimentally evaluated bioactivity is the most preferable, predicted molecules are potential basis for the design of new inhibitors. *In silico* techniques provide essential molecules which serve as baseline structures worthy of experimental exploration. Analogues or derivatives of the predicted compounds can be explored using techniques including pharmacophore modeling. If the predicted compounds are not purchasable, it offers the opportunity to synthesize. Also, compounds predicted from the robust and integrated computational study are useful to the drug discovery community since they can be tested *in vitro* and *in vivo*.

## 4. Conclusion

This study enriches current efforts in the development of novel anthelmintics by leveraging African natural-products as potential inhibitors against *Na*-GST-3. A compendium of 3182 compounds was initially filtered to remove compounds with undesirable functional groups. The pre-filtered African natural product compounds numbering 2500 in addition to five helminth GST inhibitors were virtually screened against the minimized *Na*-GST-3 ligand binding H-site. A total of 19 had reasonably good binding affinity and good docking poses in the binding pockets. The compounds formed intermolecular bonds with the known active site residues. The four identified potential leads ZINC85999636, Dammarane triterpene13, ZINC14825190 and ZINC35418176 were predicted to have anthelmintic biological activity. Moreover, the molecules showed good pharmacological profiles, had low free binding energies *via* MM-PBSA analysis and had good intermolecular interactions with the *Na*-GST-3 therapeutic target. The four molecules also had high binding affinity against other *Na*-GSTs. Ligand quality assessment showed the compounds to be meaningful templates for fragment optimization. The current study is computational which warrants further experimental evaluation to corroborate the predicted potential inhibitory activity of the molecules, including heme regulation mechanisms. The study complements the development of helminth GST inhibitors and the structures could be optimized as future therapeutic molecules.

## Supplementary Material

1

## Figures and Tables

**Table 1:** A table showing the names or IDs of the top 19 hits as well as the 5 known inhibitors, with their binding affinities in kcal/mol. The compounds were arranged in decreasing order of binding affinity.

Number	Compound Name/ZINC ID	Binding Affinities (kcal/mol)
1.	Vobtusine	−10.8
2.	ZINC13411589	−10.5
3.	Neoilexonol	−9.8
4.	ZINC28462577	−9.7
5.	Dammarane triterpene13	−9.6
6.	ZINC95486378	−9.9
7.	17alpha-hydroxycabralealactone	−9.8
8.	1(12),22(23)-tetradehydrocabralealactone	−9.8
9.	ZINC14825190	−9.6
10.	Olibanumol	−9.5
11.	ZINC34518176	−9.5
12.	ZINC00134782	−9.5
13.	ZINC95486223	−9.5
14.	ZINC95485976	−9.5
15.	4-*O*- (4″-*O*-galloyl-alpha-L-rhamnopyranosyl) ellagic acid	−9.4
16.	ZINC95486089	−9.4
17.	3′-*O*-beta-D-glucocalotropin	−9.4
18.	(−)-(R,R)-7″-*O*-methylcuspidaline	−9.4
19.	ZINC85999636	−9.3
20.	Cibacron blue	−9.9
21.	Ellagic acid	−7.8
22.	Chenodeoycholic acid	−7.8
23.	Lithocholic acid	−7.4
24.	Alizarin	−7.3

**Table 2:** The molecular interactions of selected compounds with their binding energies, interacting residues, number of bonds and lengths.

ZINC IDs/Compound Names	Binding energy (kcal/mol)	Interacting residues	Number of hydrogen bonds	Length of hydrogen bonds (Å)	Number of hydrophobic contacts	Total number of interactions
Vobtusine	−10.8	Gly13, Ala14, Ser64, Phe65, Tyr95, Glu162, Phe206	1	3.13[Arg12]	14	15
ZINC13411589	−10.5	Tyr8, Gly13, Ala14, Ser64, Phe65, Phe206	1	3.11[Tyr8]	13	14
ZINC95486378	−9.9	Tyr8, Phe9, Gly13, Phe206	1	2.96[Arg107]	9	10
Cibacron blue	−9.9	Tyr8, Phe9, Gly13, Ala14, Phe65, Tyr95, Glu162, Phe206	2	2.86 [Tyr8] 3.13 [Arg12]	15	17
17alpha-hydroxycabralealactone	−9.8	Tyr8, Gly13, Ala14, Phe206	1	3.06[Tyr95]	13	14
ZINC28462577	−9.7	Tyr8, Gly13, Ala14, Tyr95, Glu162, Phe206	3	3.07[Tyr8] 2.94[Met110] 3.18[Lys205]	9	12
Dammarane triterpene13	−9.6	Gly13, Ala14, Glu162, Phe206, Tyr95	1	3.02[Tyr95]	11	12
ZINC14825190	−9.6	Gly13, Ala14, Tyr95, Glu162	0	–	9	9
ZINC34518176	−9.5	Gly13, Ala14, Tyr95, Glu162	0	–	9	9
4-*O*-(4″-*O*-galloyl-alpha-L rhamnopyranosyl) ellagic acid	−9.4	Tyr8, Gly13, Ala14, Tyr95, Phe206	5	2.96 [Gln50] 3.12 [Gln50] 3.09 [Leu51] 2.71 [Tyr8] 3.15 [Arg12]	10	15
3’-*O*-beta-D-glucocalotropin	−9.4	Gly13, Ala14, Leu51, Ser64, Tyr95	2	2.98 [Ser64] 2.46 [Tyr95]	13	15
ZINC85999636	−9.3	Gly13, Ala14, Ser64, Phe65, Phe206, Tyr95	2	3.03[Gly13] 2.27[Tyr95]	11	13
Chenodeoxycholic acid	−7.8	Gly13, Ala14, Tyr95, Glu162	4	3.07 [Arg103] 3.19 [Arg107] 3.13 [Glu162] 3.21 [Arg12]	6	10
Ellagic acid	−7.8	Gly13, Ala14, Tyr95, Glu162	2	3.03 [Arg12] 3.01 [Tyr159]	10	12
Lithocholic acid	−7.4	Tyr8, Gly13, Ala14, Ser64, Phe65, Tyr95, Glu162	0	–	13	13
Alizarin	−7.3	Gly13, Ala14, Tyr95, Ser64	2	3.20 [Ser64] 2.86 [Tyr159]	6	8

**Table 3:** Physicochemical properties of the top 19 hits with the five standard GST inhibitors. The MW, HBD, HBA, and LogP were evaluated to predict the drug-likeness per the RO5.

No.	Compound name/ZINC ID	MW	HBD	HBA	LOGP	Violations	Druglikeness
1.	ZINC95486223	434.70	2	2	3.1109	0	Yes
2.	Vobtusine	702.88	1	7	4.8424	1	Yes
3.	ZINC13411589	448.52	0	2	7.4766	1	Yes
4.	Neoilexonol	440.71	1	2	7.2038	1	Yes
5.	Dammarane triterpene13	412.61	0	3	6.1123	1	Yes
6.	17alpha-hydroxycabralealactone	430.62	1	4	5.4512	1	Yes
7.	1(12),22(23)-tetradehydrocabralealactone	410.59	0	3	5.8883	1	Yes
8.	ZINC14825190	418.44	2	6	5.2396	1	Yes
9.	Olibanumol	186.25	2	3	0.5416	0	Yes
10.	ZINC34518176	426.72	1	1	8.0248	1	Yes
11.	ZINC00134782	344.36	0	4	5.3904	1	Yes
12.	ZINC95486089	424.70	0	1	8.3771	1	Yes
13.	3′-*O*-beta-D-glucocalotropin	532.62	3	9	2.0007	1	Yes
14.	ZINC95485976	676.75	6	9	2.3500	2	No
15.	ZINC85999636	390.47	1	4	5.7916	1	Yes
16.	ZINC28462577	538.46	5	10	5.5537	2	No
17.	ZINC95486378	630.69	5	8	8.6079	2	No
18.	(−)-(R,R)-7′-*O*-methylcuspidaline	624.77	1	8	6.7623	2	No
19.	4-*O*- (4″-*O*-galloyl-alpha-L-rhamnopyranosyl) ellagic acid	600.44	8	16	0.795	3	No
20.	Cibacron blue	771.13	4	14	2.8257	2	No
21.	Ellagic acid	302.19	4	8	1.3128	0	Yes
22.	Chenodeoycholic acid	391.56	2	4	3.1432	0	Yes
23.	Alizarin	240.21	2	4	1.8732	0	Yes
24.	Lithocholic acid	375.56	1	3	4.1724	0	Yes

**Table 4:** Two-dimensional structures, names, and molecular formulas of the four identified putative lead compounds.

ZINC ID/NANPDB ID (Compound Name)	Molecular Formula	2D STRUCTURE
ZINC85999636 ((8S)-5-Hydroxy-2,2-dimethyl-10-(3-methylbut-2-enyl)-8-phenyl-7,8-dihydropyrano[3,2-g]chromen-6-one)	C25H26O4	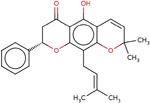
Dammarane triterpene13	C_27_H_40_O_3_	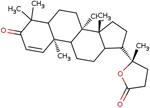
ZINC35418176 (4-[(2,3-dimethoxyphenyl)methyleneamino]-3-ethyl-1H-1,2,4-triazole-5-1H-1,2,4-triazole-5-thione)	C_13_H_16_N_4_O_2_S	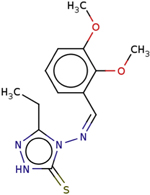
ZINC14825190 (5-Hydroxy-7-(7-hydroxy-2,2-dimethylchromen-6-yl)-2,2-dimethylpyrano[3,2-g] chromen-6-one)	C_25_H_22_O_6_	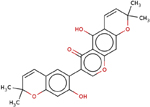

**Table 5:** Binding affinities of the eight selected compounds against the three *Na*-GSTs.

Lead Compounds	*Na*-GST-1 kcal/mol	*Na*-GST-2 kcal/mol	*Na*-GST-3 kcal/mol
Dammarane triterpene13	−8.5	−10.6	−9.6
ZINC35418176	−6.1	−6.6	−9.5
ZINC85999636	−8.8	−10.5	−9.3
ZINC14825190	−8.9	−10.8	−9.6
Cibacron blue	−9.0	−9.4	−9.9
Alizarin	−7.7	−9.0	−7.3
Chenodeoxycholic acid	−7.4	−8.7	−7.8
Lithocholic acid	− 7.2	−9.0	−7.4
Ellagic acid	− 7.2	−9.4	−7.8

**Table 6:** The average energy terms for the complexes with standard deviations in kJ/mol.

Energy Term	Dammarane Triterpene13	ZINC85999636	ZINC35418176	Cibacron Blue	ZINC14825190
Electrostatic	−110.63 ± 18.73	−28.49 ± 5.61	−2.434 ± 2.19	−235.75 ± 164.72	−5.726 ± 2.887
Van Der Waal	−235.24 ± 14.64	−225.74 ± 96.85	−210.64 ± 93.09	−161.29 ± 60.98	−244.742 ± 11.287
Non-polar	−19.31 ± 1.14	−18.07 ± 8.02	−16.54 ± 7.54	−20.577 ± 7.24	−19.879 ± 1.067
Polar	170.84 ± 26.66	84.31 ± 32.88	48.38 ± 2.81	411.71 ± 119.40	60.761 ± 11.368
Binding energy	−194.34 ± 25.55	−187.99 ± 89.49	−181.23 ± 89.22	−5.90 ± 116.17	−209.585 ± 10.842

**Table 7:** The residues contributing a large amount of energies to ligand binding. The energies were derived from the MM-PBSA calculations.

Residue	Dammarane Triterpene13 (kJ/mol)	ZINC85999636 (kJ/mol)	ZINC35418176 (kJ/mol)	Cibacron Blue (kJ/mol)	ZINC14825190 (kJ/mol)
Gly13	−1.2083	−2.7643	−2.0865	−2.3343	−3.05999
Ala14	−2.5027	−4.5778	−3.2030	−0.4831	−4.4520
Tyr95	−4.2508	−2.4909	−3.5749	−2.2440	−0.3982
Met110	−5.0135	−5.9556	−6.6696	−3.0661	−10.1475
Cys163	−4.6266	−3.8189	−2.9414	−4.6284	−4.9439

**Table 8:** Quality assessment of the selected potential lead compounds. The table shows the ligand efficiency scale (LE_Scale), ligand efficiency (LE), ligand efficiency dependent lipophilicity (LELP) and fit quality (FQ) of the selected molecules.

Compounds	LE	LE_Scale	FQ	LELP
Dammarane triterpene13	0.32	0.33619	0.95185	19.1009
ZINC85999636	0.32069	0.34673	0.9249	18.0598
ZINC35418176	0.475	0.45502	1.04392	16.8943
ZINC14825190	0.3097	0.3259	0.95029	16.9196
