# Supplementary material for: Computer-aided identification of potential inhibitors against Necator americanus glutathione S-transferase 3
Source: Inform Med Unlocked. Author manuscript; Available in PMC 2022 Dec 23. (PMC9784411; doi:10.1016/j.imu.2022.100957)
Supplement: 1 [file NIHMS1808136-supplement-1.docx]

SUPPLEMENTARY FILES


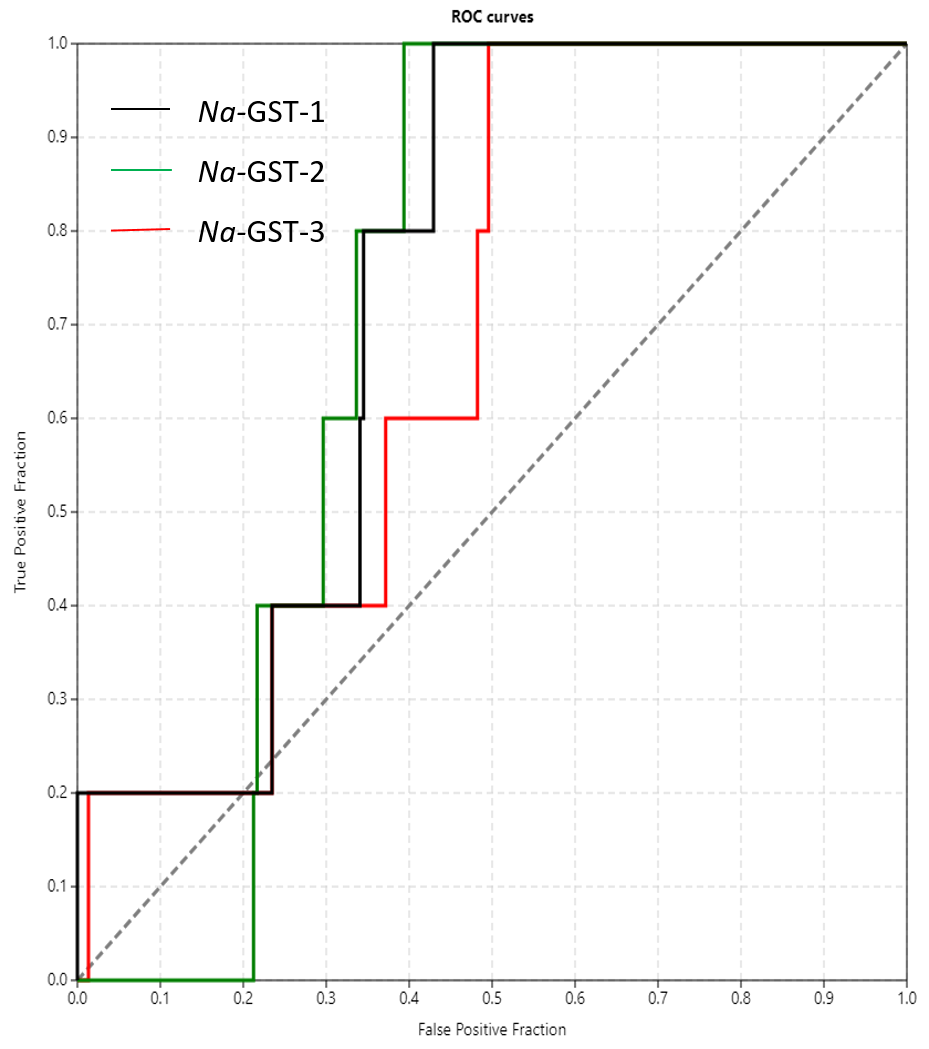


Supplementary Figure 1: ROC curve generated by screening 225 decoys alongside five inhibitors against the *Na-*GSTs receptors. Reasonably good AUC values of the ROC curves of 0.681, 0.709, and 0.731 were obtained for *Na-*GST-1, *Na-*GST-2, and *Na-*GST-3, respectively.


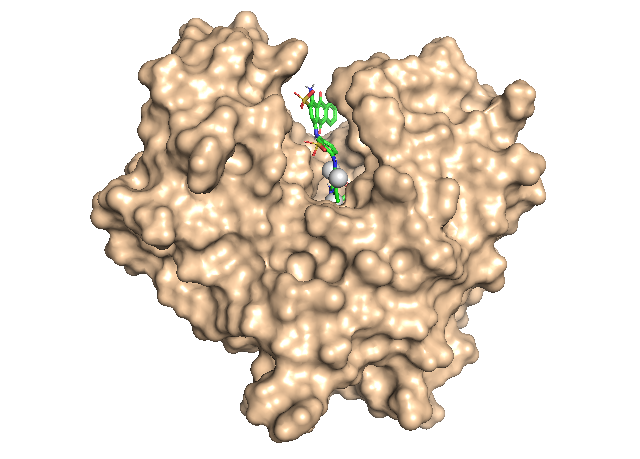


Supplementary Figure 2: Protein-ligand complex of cibacron blue and *Na*-GST-3. The protein is represented as a surface whereas the ligand is represented as sticks. The ligand as shown does not dock deep in the binding pocket.


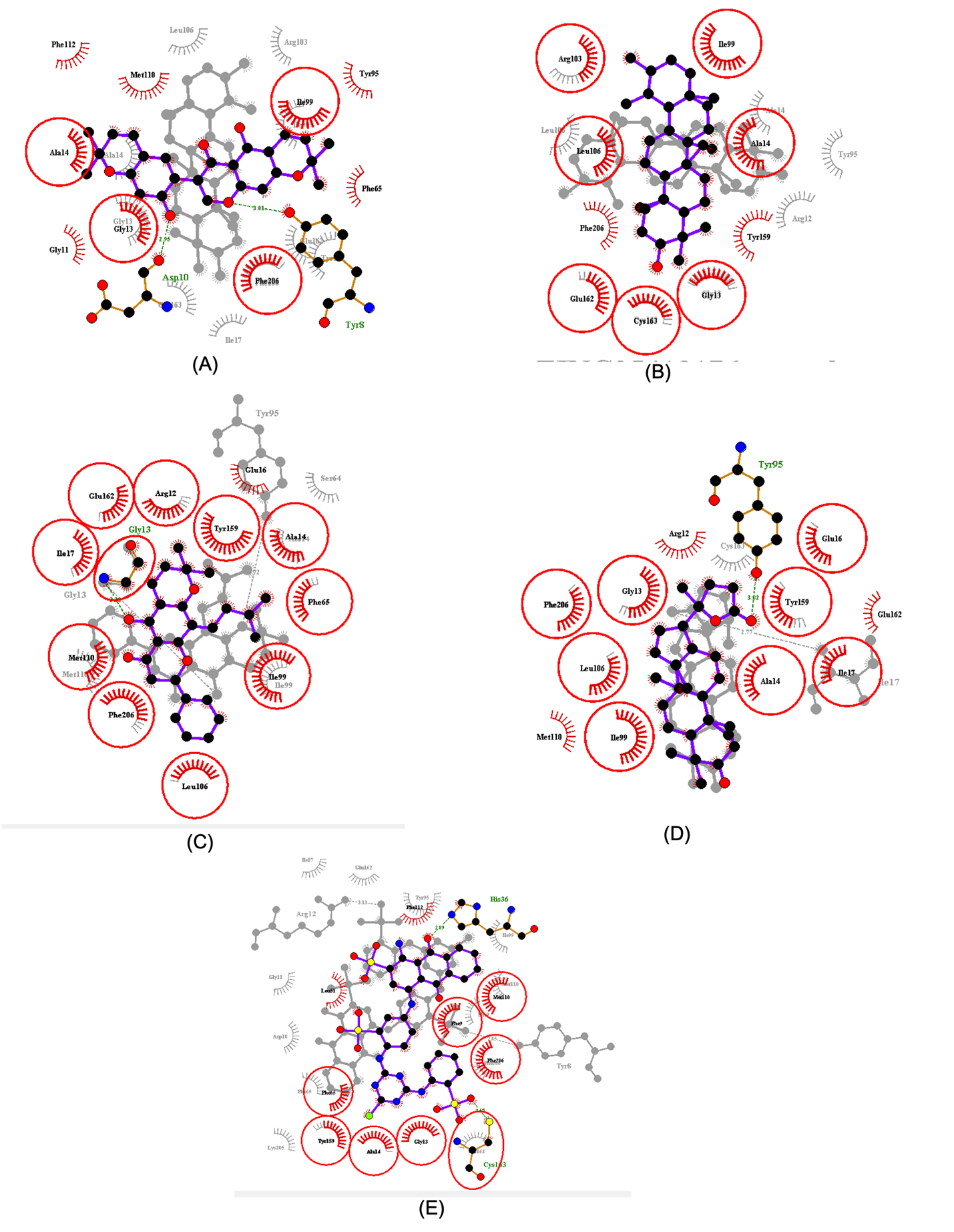


Supplementary Figure 3: Analysis of the pre- and post-MD binding poses for a) ZINC14825190, b) ZINC35418176, c) ZINC85999636, d) cibacron blue, and e) dammarane triterpene13 complexes


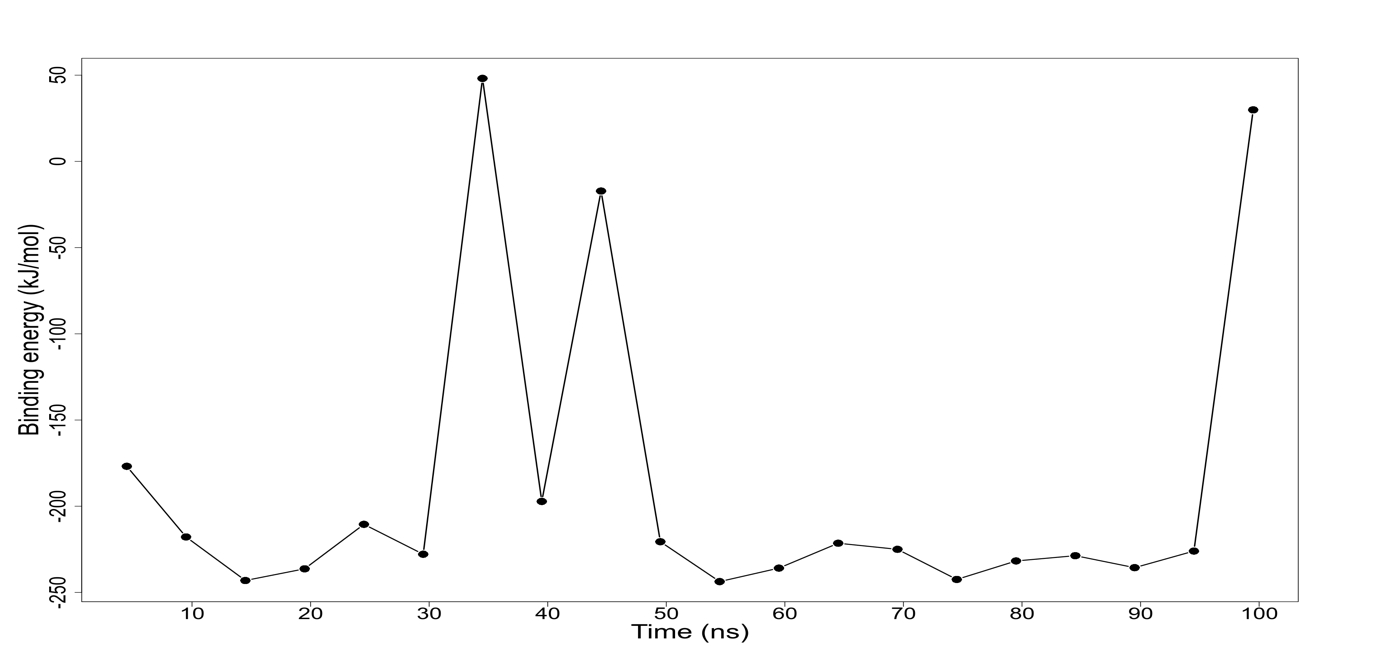


a


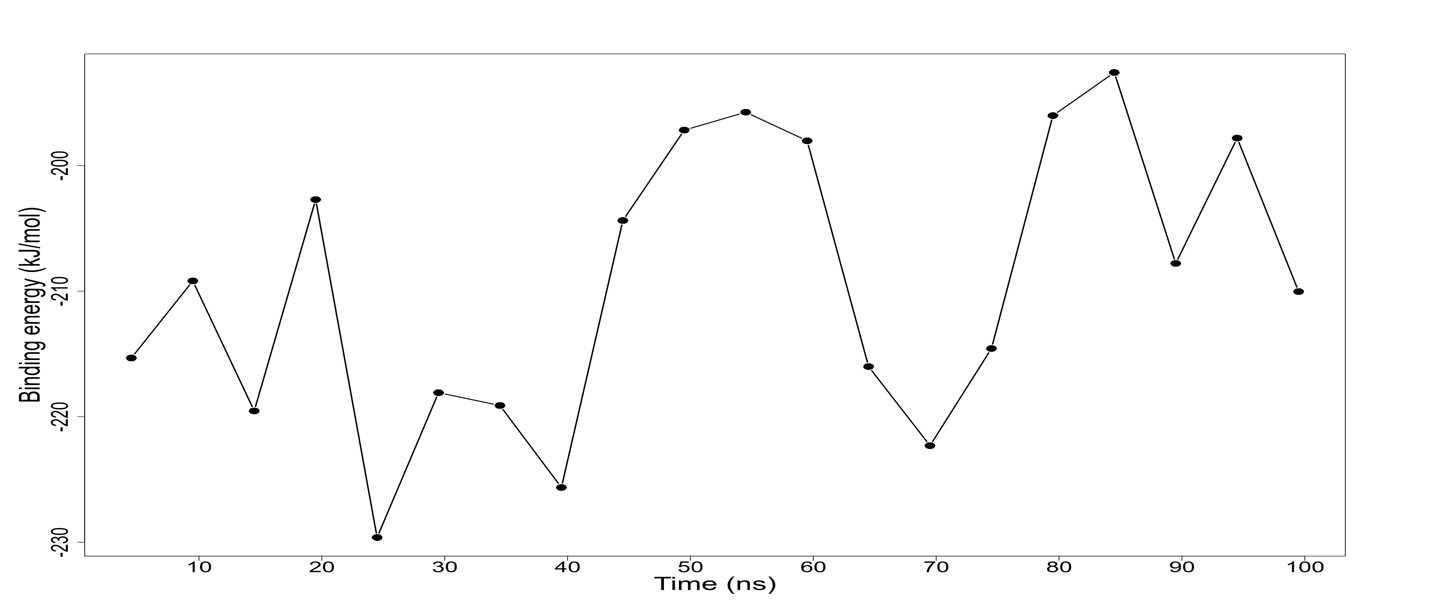


b


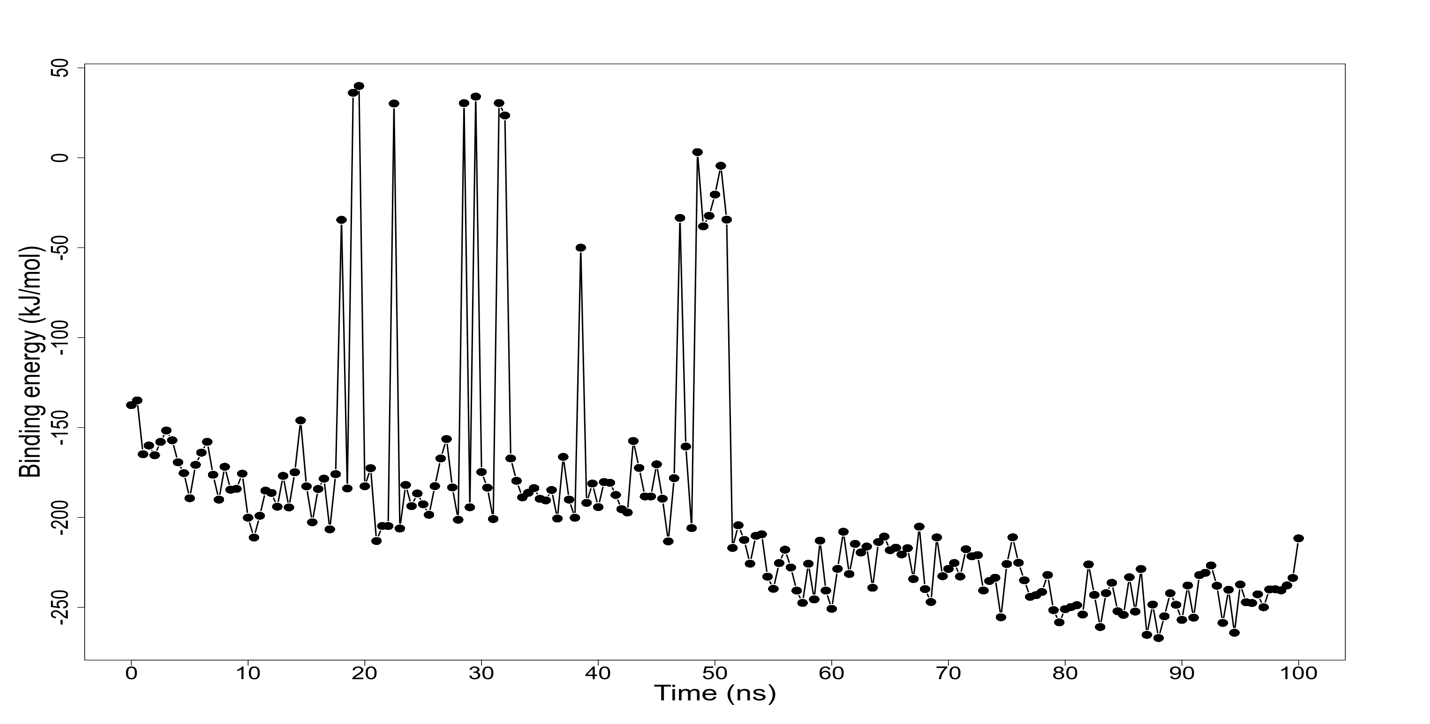


c


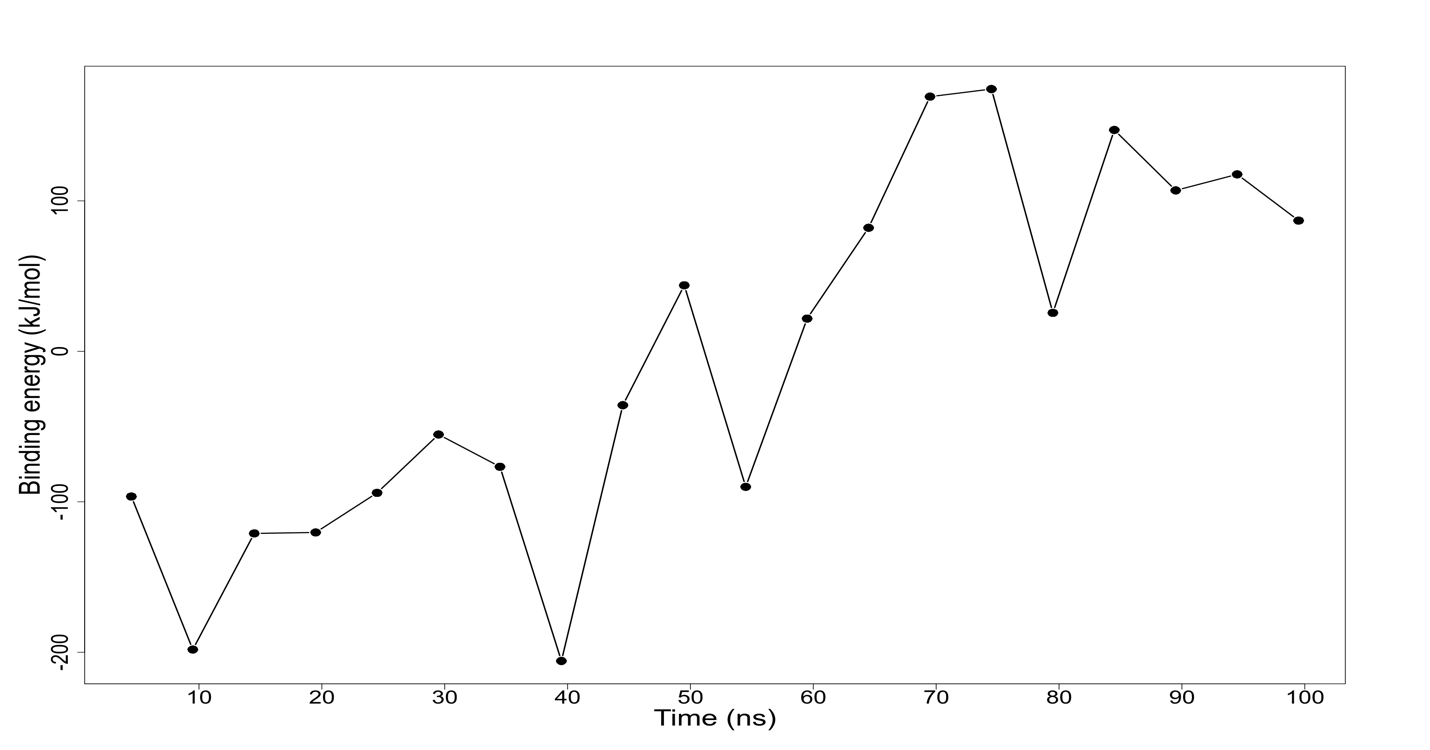


d

Supplementary Figure 4: Molecular mechanics Poisson-Boltzmann surface area (MM-PBSA) binding energy plot of (a) ZINC85999636, (b) ZINC14825190, (c) ZINC35418176, and (d) cibacron blue complexes**.** Binding energy (kJ/mol) versus time graph over 100 ns simulation


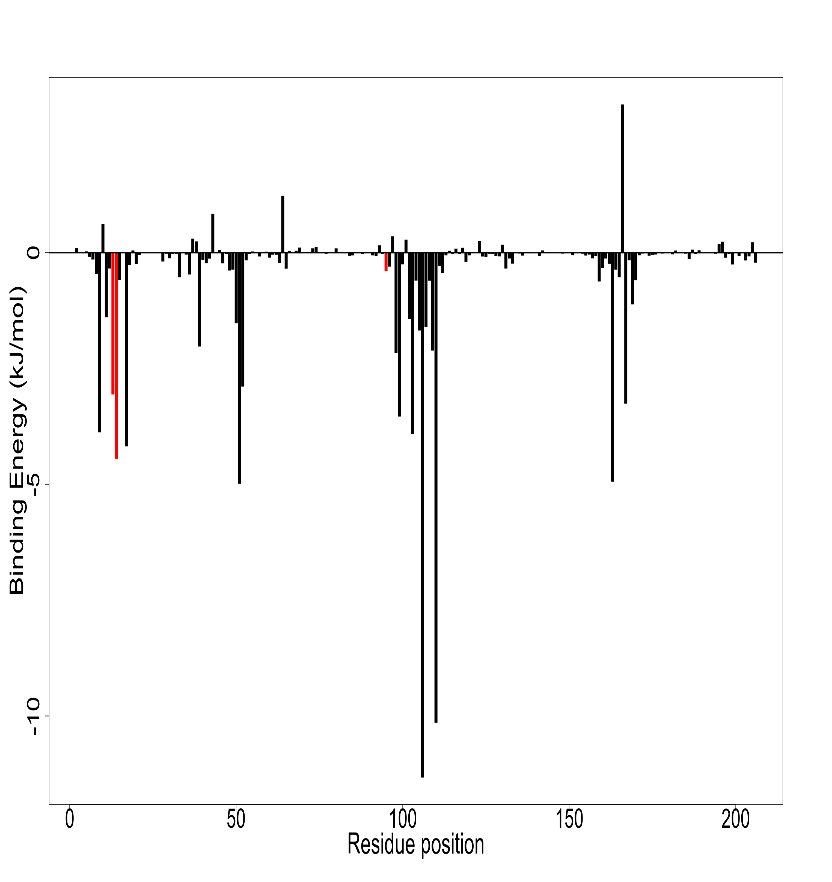

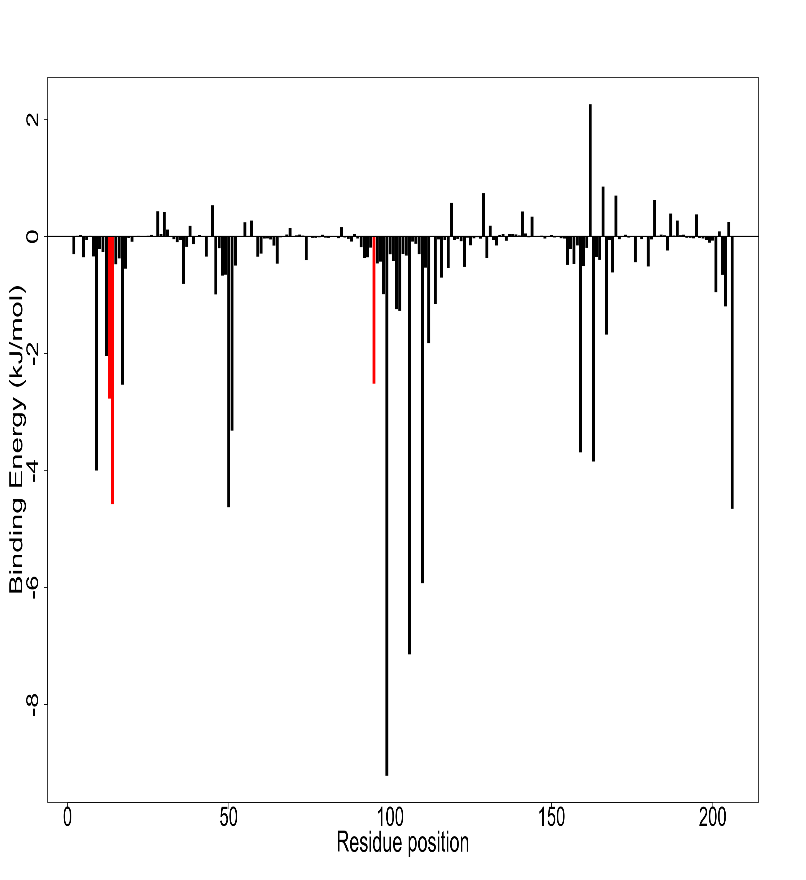


b

a


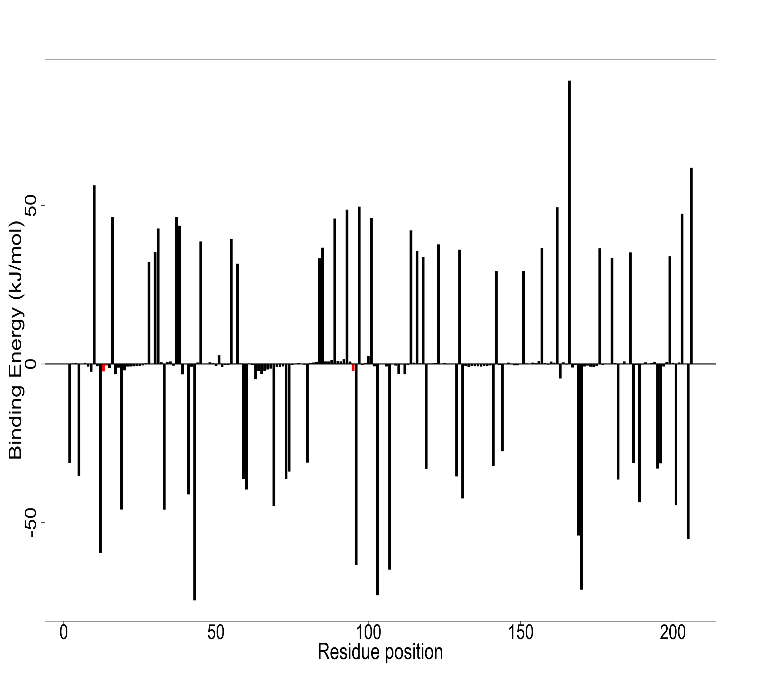

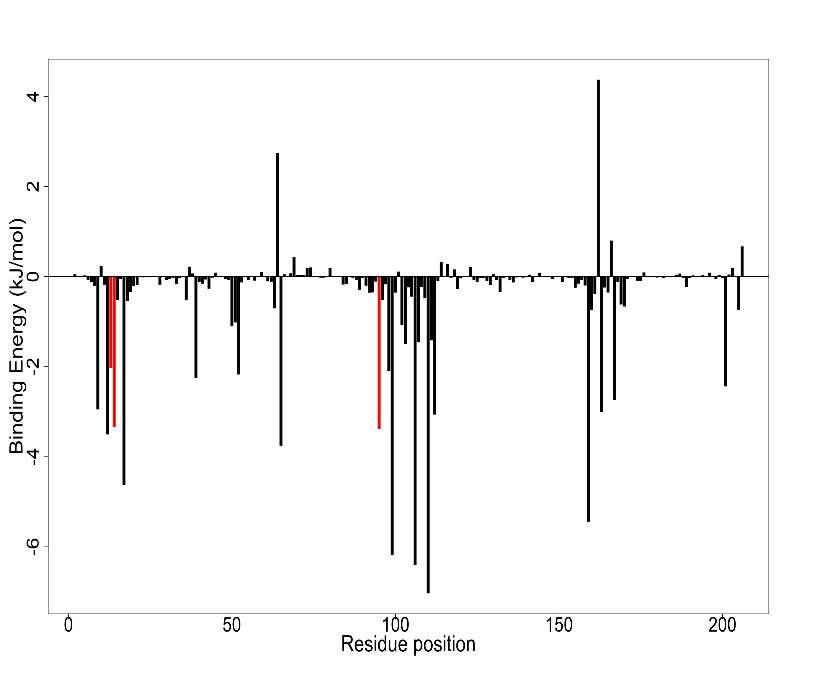


d

c

Supplementary Figure 5: Molecular mechanics Poisson-Boltzmann surface area (MM-PBSA) plots of binding free energy contribution per residue of (a) ZINC85999636, (b) ZINC14825190, (c) ZINC35418176, and (d) cibacron blue complexes. The total binding energy contribution of the suggested critical residues are colored red.

**Supplementary Table 1**: Predicted anthelmintic activity using PASS [49]. Probability of activity (Pa) and the probability of inactivity (Pi) of the selected nine hits. When the Pa>Pi, the pharmacological profiles of the hits are worth probing further experimentally.

| **ZINC ID/ COMPOUND NAME** | **Pa** | **Pi** | **Pa > Pi** |
| --- | --- | --- | --- |
| ZINC85999636 | 0.540 | 0.005 | Yes |
| ZINC14825190 | 0.486 | 0.008 | Yes |
| ZINC28462577 | 0.457 | 0.025 | Yes |
| ZINC00134782 | 0.249 | 0.059 | Yes |
| 4-O-(4’’-O-galloyl-alpha-L-rhamnopyranosyl) ellagic acid | 0.2471 | 0.164 | Yes |
| ZINC95486378 | 0.247 | 0.060 | Yes |
| Dammarane triterpene13 | 0.206 | 0.085 | Yes |
| 1(12),22(23)-tetradehydrocabralealactone | 0.194 | 0.096 | Yes |
| 17alpha-hydroxycabralealactone | 0.184 | 0.108 | Yes |

**Supplementary Table 2**: Pharmacokinetics of top twenty hits as well as well as the five GST inhibitors showing gastrointestinal absorption (GI), permeability-glycoprotein (Pgp), CYP3A4 inhibition, mutagenicity, tumorigenicity, irritant and reproductive effects.

| Name | GI absorption | Pgp substrate | CYP3A4 inhibitor | Mutagenic | Tumorigenic | Reproductive Effect | Irritant |
| --- | --- | --- | --- | --- | --- | --- | --- |
| Vobtusine | High | Yes | No | None | None | None | None |
| ZINC13411589 | Low | No | No | None | None | None | None |
| Neoilexonol | Low | No | No | None | None | None | None |
| ZINC28462577 | Low | No | No | None | None | High | None |
| Dammarane triterpene13 | High | No | No | None | None | None | None |
| ZINC95486378 | Low | No | No | High | High | High | High |
| 17alpha-hydroxycabralealactone | High | No | No | None | None | High | None |
| 1(12),22(23)-tetradehydrocabralealactone | High | No | No | None | Low | None | High |
| ZINC14825190 | High | No | No | None | Low | High | None |
| Olibanumol | High | No | No | None | None | None | None |
| ZINC35418176 | Low | No | No | None | None | None | None |
| ZINC00134782 | High | No | Yes | None | None | None | None |
| ZINC95486223 | High | No | No | None | None | None | None |
| ZINC95485976 | Low | No | No | High | None | None | High |
| 4-O-(4’’-O-galloyl-alpha-L-rhamnopyranosyl) ellagic acid | Low | No | No | None | None | None | None |
| ZINC95486089 | Low | No | No | None | None | None | None |
| 3’-O-beta-D-glucocalotropin | High | Yes | No | None | None | None | None |
| (-)-(R,R)-7’-O-methylcuspidaline | High | No | No | None | None | None | None |
| ZINC85999636 | High | No | Yes | None | None | High | None |
| Alpha-amyrin | Low | No | No | None | None | None | None |
| Cibacron blue | Low | No | No | High | High | High | High |
| Ellagic acid | High | Yes | No | None | None | None | None |
| Chenodeoycholic acid | High | No | Yes | None | None | None | None |
| Lithocholic acid | High | No | No | None | None | None | None |
| Alizarin | High | Yes | No | High | None | None | High |
